# Degradation of perineuronal nets in hippocampal CA2 explains the loss of social cognition memory in Alzheimer's disease

**DOI:** 10.1002/alz.70813

**Published:** 2025-10-22

**Authors:** Lata Chaunsali, Jiangtao Li, Erik Fleischel, Courtney E. Prim, Izabela Kasprzak, Shan Jiang, Silky Hou, Miguel Escalante, Elise C. Cope, Michelle L. Olsen, Bhanu P. Tewari, Harald Sontheimer

**Affiliations:** ^1^ Department of Neuroscience University of Virginia School of Medicine Charlottesville Virginia USA; ^2^ School of Neuroscience Virginia Tech Blacksburg Virginia USA

**Keywords:** Alzheimer's disease, CA2, extracellular matrix, matrix metalloproteinases, perineuronal nets, social memory

## Abstract

**INTRODUCTION:**

Loss of social cognition memory impairs Alzheimer's disease (AD) patients to recognize family members, friends, and caregivers. We investigate the role of perineuronal nets (PNNs), specialized coats of extracellular matrix around hippocampal CA2 neurons in AD‐associated social memory impairments.

**METHODS:**

We utilized 5XFAD mouse model of AD and employed immunohistochemistry, microscopy, bulk RNA‐sequencing, animal behavior, gene‐knockout, and drug‐treatment approaches.

**RESULTS:**

AD mice showed profound disruption of CA2 PNNs with concomitant impairment of social cognition memory. Genetic or enzymatic CA2 PNN disruption in wild‐type mice mimicked these impairments. Transcriptomic analysis shows upregulation of PNN‐cleaving matrix metalloproteinases (MMP) in AD mice causing disequilibrium of PNN synthesis and remodeling. Chronic inhibition of MMPs retains CA2 PNN and delays social memory impairments in 5XFAD mice.

**DISCUSSION:**

AD‐associated social memory impairments are caused by loss of CA2 PNNs. Inhibition of PNN proteolysis by MMPs preserves social memory, suggesting PNN as a promising therapeutic target.

**Highlights:**

- Perineuronal nets (PNNs) are disrupted in the CA2 area of the hippocampus in 5XFAD Alzheimer's disease (AD) mice at 6 months of age and beyond.
- Social memory deficits in 5XFAD mice coincide with the disruption of CA2 PNNs and PNN loss alone is sufficient to cause loss of social memory.
- Bulk RNA sequencing of hippocampal CA2 tissue reveals alterations in PNN remodeling enzymes.
- Inhibition of matrix metalloproteinase (MMP) activity with GM6001 prevents PNN disruption and protects against social memory deficits in the 5XFAD AD mouse model.

## 1 BACKGROUND

Alzheimer's disease (AD) is the predominant form of dementia afflicting ∼55 million people worldwide with an anticipated increase of 35% in the next five years.^1^, ^2^ The disease progresses from subtle symptoms including a gradual decline in cognitive functions such as memory, attention, language, and social behaviors, and evolves to its severe forms eventually causing profound memory loss, difficulty in communicating, impaired judgment, and inability to recognize loved ones.^3^ These symptoms coincide with histopathological abnormalities in the brain, including deposition of extracellular β‐amyloid (Aβ) plaques and intracellular neurofibrillary tangles of hyperphosphorylated tau, loss of neurons and synapses, neuroinflammation, and oxidative stress.

One of the emerging pathological signatures of AD brains^4^ is the alteration in the brain extracellular matrix (ECM) and its condensed form known as perineuronal net (PNN). The lattice‐like perforated coats of PNNs are composed of an interwoven network of hyaluronan (HA), link proteins, glycoproteins, and several chondroitin sulfate proteoglycans (CSPGs) such as aggrecan (Acan), brevican, and neurocan. Although PNNs predominantly coat inhibitory parvalbumin (PV) expressing neurons throughout the brain, in the hippocampal CA2 area, PNNs enclose both excitatory pyramidal and inhibitory PV neurons. The CA2 area is anatomically^5^ and molecularly^6^, ^7^ distinct from the remaining hippocampal pyramidal neurons, and molecular markers such as Purkinje cell protein 4 (PCP4) and Regulator of G‐protein Signaling 14 (RGS14) are commonly used to identify CA2 neurons.

Several AD models and human AD brains show a progressive deterioration of the PNNs around inhibitory neurons and altered expression of ECM components in several key brain areas involved in memory and cognition including hippocampus, subiculum, and entorhinal cortex.^8^, ^9^, ^10^, ^11^ These changes are often linked to neuroinflammation^8^ that triggers overexpression of proteolytic enzymes including matrix metalloproteinases (MMPs), a disintegrin and metalloproteinase with thrombospondin motifs (ADAMTSs), and their regulators, such as tissue inhibitors of metalloproteinases (TIMPs).^8^, ^12^, ^13^

PNNs are classically known for stabilizing synapses thereby preventing synaptic plasticity and preserving memories.^14^ We recently reported that PNNs are also pivotal for maintaining the structure and function of tripartite synapses and showed that in the 5XFAD model of AD, disrupted cortical PNNs are associated with altered tripartite synapses.^15^ Based on these and other studies reporting altered PNNs in AD, we hypothesized that PNN disruption may be a key pathological event in AD etiology^16^ that can destabilize synapses, expose them to inflammation‐induced stressors, and consequently synaptic dysfunction impairs memory and cognitive functions.

Social recognition memory, which enables individuals to distinguish between familiar and novel conspecifics and learn from social interactions, is impaired in AD patients and animal models.^17^, ^18^ The clinical onset of social memory deficits in AD is often subtle, beginning with difficulty recognizing familiar faces, recalling names, and remembering past interactions. As the disease progresses, individuals may struggle with interpreting social cues, maintaining relationships, and recognizing loved ones, leading to increased social withdrawal and isolation. These deficits are believed to arise due to early dysfunction in brain regions such as the hippocampus and prefrontal cortex, which are critical for social cognition and memory.^19^, ^20^ Recent studies have shown alterations in the brain ECM and PNNs in these regions in AD brains;^8^ however, whether and how these changes are associated with the decline in social memory remains unknown.

In the present study, we report a pronounced loss of PNNs around CA2 pyramidal neurons in the 5XFAD model of AD, which precisely coincides with the loss of social memory while other hippocampus‐dependent memories remain intact. We establish a causal relation between CA2 PNN and social memory by reporting loss of social memory upon genetic and enzymatic disruptions of the CA2 PNNs in control mice. The transcriptomic analysis of the CA2 area from AD brains suggests that an imbalance of PNN synthesis and remodeling mechanisms, predominantly the overexpression of multiple MMPs contributes to PNN loss. Finally, we show that treating 5XFAD mice at the presymptomatic stage with a broad‐spectrum MMP inhibitor, ilomastat or GM6001, can prevent PNN loss and consequently delay the development of social memory dysfunction in 5XFAD mice. Overall, our study not only establishes a causal relationship between AD‐associated CA2 PNN disruption with the loss of social memory but also provides compelling evidence that pharmacological inhibition of PNN proteolysis can prevent social memory loss, suggesting PNNs as a potential therapeutic target.

## 2 METHODS

### 2.1 Mice

All animal procedures were approved and performed according to the ethical guidelines set by the University of Virginia Institutional Animal Care and Use Committee (IACUC). Mice were housed in groups of five in a facility in a 12‐h light‐dark cycle with controlled temperature (21 ± 1.5°C) and humidity (50 ± 10%) and had access to food and water ad libitum. We obtained 5XFAD mice (B6SJL‐Tg (APPSwFlLon, PSEN1*M146L*L286V) 6799Vas/ Mmjax, strain 034840‐JAX) from the Jackson Laboratory and backcrossed for five generations to C57Bl/6J (000664‐JAX) wild‐type (WT) mice to obtain an incipient congenic line on a C57Bl/6J genetic background for experiments. The 5XFAD mice harbor five mutations across two human transgenes, amyloid precursor protein (APP) and presenilin‐1 (PSEN1), as described in detail elsewhere.^21^ We obtained J20 mice (B6.Cg‐Tg(PDGFB‐APPSwInd)20Lms/2J), strain 034836‐JAX) from Jackson Laboratory. hAPPJ20 line overexpresses the hAPP (human *APP*) minigene with Swedish (K670M/N671L) and Indiana (V717F) mutations under control of the *PDGFB* promoter, were obtained in C57BL/6J background. 3XTg mice (#034830‐JAX) brain slices were a generous gift from Dr. Heather Ferris at UVA. We received C57BL/6N‐Acan^tm1c(EUCOMM)Hmgu/H^ (European Mouse Mutant Archive stock EM:10224) from EUCOMM (UK Research & Innovation, Mary Lyon Center). The heterozygous mice were bred together to generate *Acan^fl/fl^* mice as described previously.^15^ Unless stated otherwise, we used both male and female 5XFAD+ and age‐matched WT (5XFAD‐) mice in our immunohistochemical (IHC) studies, whereas behavioral studies were conducted using only male mice following previous studies.^22^ The researchers were blinded to genotype and treatment groups during the analysis of histological and behavioral data. All mice were genotyped to confirm the transgene expression or knockout before experimental use.

### 2.2 Intracranial surgeries and injections

#### 2.2.1 ChABC injection

Chondroitinase ABC (ChABC) is a bacterial‐derived (*Proteus vulgaris*) enzyme (C3667‐10UN, Sigma‐Aldrich) that cleaves sulfated sugar chains of the PNNs and is commonly utilized to disrupt PNN in vitro and in vivo.^23^ We dissolved ChABC in sterilized phosphate buffered saline (PBS) (50 mU/µL) followed by bilaterally injecting ChABC or control enzyme penicillinase (PNase, 50 U/mL, Sigma) in mice. We anesthetized 7‐ to 8‐week‐old C57Bl/6J mice with 2%–5% isoflurane, provided analgesia (0.1 mg/kg buprenorphine and 5 mg/kg carprofen i.p.), and head fixed them to a stereotaxic apparatus (David Kopf Instruments) followed by a midline scalp incision and a 0.5‐mm burr hole. We used −1.8 AP, ± 2.2 ML, and −1.7 DV coordinates to target CA2 in all the mice. Subsequently, 50 nL solution was bilaterally injected using a 10‐µL neuros syringe (Model 1701, 65460‐06, Hamilton) and 33‐gauge needle (65461‐02, Hamilton) at an infusion rate of 20 nL/min. To ensure specificity of the used reagent, we compared the effect of heat‐inactivated ChABC with the PNase injected control, both of which showed no disruption of PNNs (Figure S1). Sham control mice were injected with PNase (50 U/mL, Sigma) with an identical procedure. Subsequently, mice were allowed to recover on a heating pad until mobile. We monitored mice daily for up to 5 days after surgery. Body weight was measured for 3 consecutive days after surgery, and all mice were perfused post‐behavior on days 5 and 14 after injection. PNN disruption was confirmed by the loss of Wisteria floribunda agglutinin (WFA) staining in CA2 (Figure S1A,B) in combination with the appearance of CS stub 2B6 staining (Figure S1A,C). Cortical areas proximal to the injection site also showed a low WFA intensity (Figure S1A,D); however, cortical areas distal to the injection site showed no change in WFA intensity (Figure S1E).

**RESEARCH IN CONTEXT**

1. **Systematic review**: We reviewed a century of research on perineuronal nets (PNNs) and their role in brain development, memory, and synaptic stability. Separate studies highlight the CA2 region's role in social cognition memory, particularly in recognizing social partners. However, the link between PNNs and social memory in CA2 remains unexplored.
2. **Interpretation**: Our findings suggest that PNN degradation around CA2 pyramidal neurons contributes to social memory impairment in Alzheimer's disease (AD). This process, driven by upregulated matrix metalloproteinases (MMPs), links extracellular matrix remodeling to cognitive decline.
3. **Future directions**: We propose that inflammation‐induced enzyme activity drives specific memory loss in AD. Targeting MMPs with inhibitors may offer a therapeutic strategy to prevent social memory deficits.

#### 2.2.2 SynCreAAV injection

To genetically knock out PNN in the CA2 area, we injected pENN.AAV.hSyn.HI.eGFP‐Cre.WPRE.SV40 (Addgene, 105540‐AAV9) in 7‐ to 8‐week‐old *Acan^fl/fl^* and *Acan^wt/wt^* (control) mice targeting CA2 area of hippocampus.^22^, ^24^ In brief, AAV9 (2.7 × 10^13^ vg per mL) was diluted in PBS to achieve 1 × 10^13^ vg per mL concentration and 50 nL was injected in each hemisphere using −1.8 AP, ± 2.2 ML, and −1.7 DV coordinates with 20 nL/min infusion rate as described above. We performed social memory behavior on these mice after 8 weeks of AAV incubation. Subsequently, we fixed mice brains by transcardial perfusion to perform IHC.

### 2.3 Tissue isolation by CA2 punching and RNA isolation

To isolate the CA2 area of the hippocampus for the bulk sequencing, we prepared 500 µm acute coronal brain slices as described previously.^25^ Slices were transferred to a Petri dish containing cold carbogen‐bubbled (95% O_2_ + 5% CO_2_) cutting solution. Slices with hippocampus were visualized under a dissection microscope, and the CA2 area was anatomically identified by the neurons with larger cell bodies than CA1 neurons and with an intermediate cell density between loosely packed CA3 and more densely packed CA1 neurons.^26^ Using a tissue puncher (Ted Pella #15116‐1 ID 1 mm), we isolated the CA2 area and snap‐froze it. After punching, we stained the remaining slices with WFA and PCP4 to confirm that the CA2 area was missing in the punched slices. All samples were immediately lysed and homogenized using plastic tissue grinders before being stored in pre‐chilled tubes at −80°C for a minimum of 24 h. RNA isolation was carried out on thawed samples using a TRIzol Plus RNA Purification Kit (Cat#12183555, Invitrogen).

### 2.4 Bulk sequencing

Following purification and nanodrop sample verification, samples were sent to the Yale Center for Genome Analysis (New Haven, CT), where they underwent mRNA (Poly A) library preparation and sequencing on an Illumina NovaSeq. FastQC v0.11.5 was used to assess the quality of each sample. Cutadapt v3.4 was used to trim adapters. Reads were aligned to the mouse mm39 reference genome using STAR v2.7.9a, and gene read counts were generated using featureCounts v2.0.6.

### 2.5 Transcriptomics analysis

Analyses were performed in R Statistical Software (v4.4.1 R Core Team 2024). Normalization, visualization, and differential analysis were conducted using DESeq2 v1.44.0. Genes with an adjusted *p*‐value < 0.05 and absolute log2 fold‐change > 1.00 were considered differentially expressed. GOplot package in R was used to generate gene ontology (GO) term circle plots.^27^

### 2.6 Behavioral experiments

#### 2.6.1 Direct social interaction test

To assess social memory, we used a modified direct social interaction test protocol used previously.^22^ Mice were habituated in the behavior room by the experimenter for 3 days before the day of the experiment, daily for 5 min to reduce anxiety. The mice were transported to the behavior room an hour before on the experiment day to get adapted to the environment and kept under low light before and during the experiment. We used (35 × 35 × 35 cm) open field box for this experiment. On the test day, mice were first habituated to the box for 5 min. Then the test mouse was introduced to a novel mouse (Novel 1) with whom the test mouse had never interacted. Subsequently, the test mouse was placed back in the home cage. After an hour of the first trial, the test mouse again interacted with the same mouse who was previously Novel 1, however, became familiar in the second trial. After the second trial, the test mouse was again placed back in the home cage for 1 h. On the third trial, the test mouse interacted with another novel mouse (Novel 2). Nose‐to‐nose, nose‐tail, anogenital sniffing, allogrooming, and the following initiated by the test mouse were used to qualify for mouse interaction. The time taken by the test mouse interacting with the encountered mice was used for counting the interaction time.

#### 2.6.2 Object recognition memory

To assess object recognition memory, we used a modified object recognition memory protocol previously used.^28^ The mice were transported to the behavior room an hour before the experiment day to get adapted to the environment and kept under low light before and during the experiment. We used (35 × 35 × 35 cm) open field box for this experiment. On the test day, first, the mice were habituated to the box for 10 min. This test consists of two phases: familiarization and test. During the first familiarization phase, two identical objects were used and kept in the arena ∼5 cm away from the wall in the two corners of the arena. Mice were then placed in the arena to familiarize themselves with the objects and arena for 10 min. Time spent investigating is considered a familiarization / training phase. After this phase mice were kept in their home cage for 30 min. During this time one object stayed in the same location whereas another object was replaced by a new object. After 30 min of interval mice were placed again in the arena for 10 min. Exploration of an object was recorded when the mouse approached an object and touched it with its vibrissae, snout, or forepaws and was measured using video tracking software (Noldus Ethovision XT). The preference for either the novel or familiar object was calculated as the percentage of time the mouse spent with one object divided by the total time the mouse spent investigating either object. The discrimination score was calculated by calculating (Time spent with novel object—Time spent with familiar object) / (Time spent with novel object + Time spent with familiar object).

### 2.7 Tissue preparation and immunohistochemistry (IHC)

Mice were deeply anesthetized by i.p. injecting a mixture of ketamine and xylazine (100 and 10 mg/kg, respectively) followed by transcardial perfusion with PBS and cold 4% paraformaldehyde (PFA). Dissected brains were moved to 4% PFA at 4°C for overnight. The next day, brains were transferred to PBS and kept at 4°C until sectioning was performed. 50‐µm‐thick coronal sections were cut by using Pelco EasiSlicer from Ted Pella vibratome. Sections were either used for IHC or stored at −20°C in a custom‐made storage medium (10% (v/v) 0.2 mM phosphate buffer, 30% (v/v) glycerol, 30% (v/v) ethylene glycol in deionized water, pH 7.2–7.4) for future uses. For IHC, −20°C stored sections were sorted in 24‐well plates and washed in PBS three times for 5 min each. Sections were permeabilized and blocked for 2 h at room temperature (RT) in blocking buffer (0.5% Triton X‐100 and 10% goat serum in PBS). Subsequently, we incubated sections with primary antibodies in diluted blocker buffer (1 blocking buffer: 3 PBS) for 16–18 h at 4°C on a rocker. The details of various antibodies and labelling reagents are given in Table S1. Next, we incubated sections with appropriate secondary antibodies in diluted blocking buffer overnight at 4°C in the dark. Further, the sections were rinsed with PBS and were mounted on glass slides (Fisherfinest 25 × 25 × 1, 12‐544‐2) covered with cover glass, and the edges of the slides were sealed with nail polish. All antibodies used in this study were validated either by vendors and/or by many published studies.

### 2.8 Confocal microscopy and image analysis

Confocal micrographs were acquired using an Olympus FV 3000 confocal microscope and analyzed using ImageJ. We utilized several different objective lenses, including ×10 (air), ×20 (air), ×40 (air), ×60 (oil), or ×100 (oil) with a range of optical zoom as needed by the experiment. We tuned the acquisition parameters including laser power, photomultiplier tube (PMT) gain, and offset, to accommodate the full range of signal as reflected by a few under‐saturated and a few saturated pixels in the images. Subsequently, we acquired 12‐bit images and minimally adjusted the acquisition settings.

We analyzed CA2 PNNs with a modified image analysis protocol as described previously.^15^ In brief, we used the WFA‐covered area and intensity of the WFA‐covered area in the PCP4‐stained CA2 area as analysis metrics for assessing PNN in various experimental groups. To assess the PNN‐covered area, we used single‐plane confocal micrographs of the hippocampus containing the entire CA2 area. To delineate the CA2 area, we drew a large, rotated rectangular region of interest (ROI) covering all PCP4‐expressing neurons in the CA2 and saved it for reference and further analysis. Subsequently, we generated binary representations of PCP4, NeuN, and WFA signals using inbuilt auto thresholding functions (OTSU or Triangle) in ImageJ. Using Boolean operations in ImageJ, we computed the binary areas of WFA, PCP4, and NeuN within the rectangle area covering the CA2. The expression of PCP4 remained unaltered in all studied age groups (Figure S2); therefore, we used PCP4 area as an internal control, and normalized the WFA area with the PCP4 area within the CA2 area rectangle and used data points to plot in graphs. Similarly, we computed the WFA intensity within the CA2 area rectangle.

To assess the neuronal density in the CA2 area, we drew an ROI encompassing the PCP4‐covered area and counted the total number of NeuN‐expressing neurons in single‐plane images. Next, we normalized the cell count with the CA2 area ROI to obtain cell density in CA2. To assess PV cell density in the CA2 area, we used ∼20 µm‐thick z‐stacks and counted the total number of PV‐expressing neurons followed by normalizing it with the PCP4‐covered CA2 volume.

### 2.9 GM6001 treatment

GM6001 or ilomastat (ApexBio Cat# A4050) is a broad‐spectrum MMP inhibitor (Ki: 0.4 nM for MMP‐1, 27 nM for MMP‐3, 0.5 nM for MMP‐2, 0.1 nM for MMP‐8, and 0.2 nM for MMP‐9). We prepared GM6001 suspension of 100 mg/kg in 4% carboxymethylcellulose (CMC) in saline as described in detail in our recent study.^24^ Mice in the control group received 4% CMC without GM6001. Mice were intraperitoneally (i.p.) injected once daily for 1 month, and subsequently, brains were collected for further experiments.

### 2.10 Statistical analysis

Data analysis was performed using GraphPad Prism 9. Data in the bar graphs are expressed as mean ± SEM unless stated otherwise in the figure. Dots in the bars represent individual data points. The sample size was based on relevant studies published by us and others.^8^, ^15^, ^22^, ^29^ Data distribution was sufficiently normal and variance within groups was sufficiently similar, therefore, we used parametric tests for statistical analysis. Experimental designs with two treatment groups were analyzed by two‐tailed unpaired *t*‐test unless stated otherwise in figure legends. Experimental designs with more than two groups were analyzed using one‐way or two‐way analysis of variance (ANOVA) followed by Tukey's or Sidak's post hoc multiple comparison tests. The statistical details including mean, SEM, n, test statistics, and post‐hoc comparisons are summarized in Tables S2 and S3. RNAseq data analysis is summarized in Table S4. Statistically significant differences between groups are shown in graphs as *p < 0.05, **p < 0.01, ***p < 0.001, and ****p < 0.0001. No data were excluded.

## 3 RESULTS

### 3.1 PNNs are disrupted in the hippocampal CA2 area in the 5XFAD mouse model of AD

**FIGURE 1:**
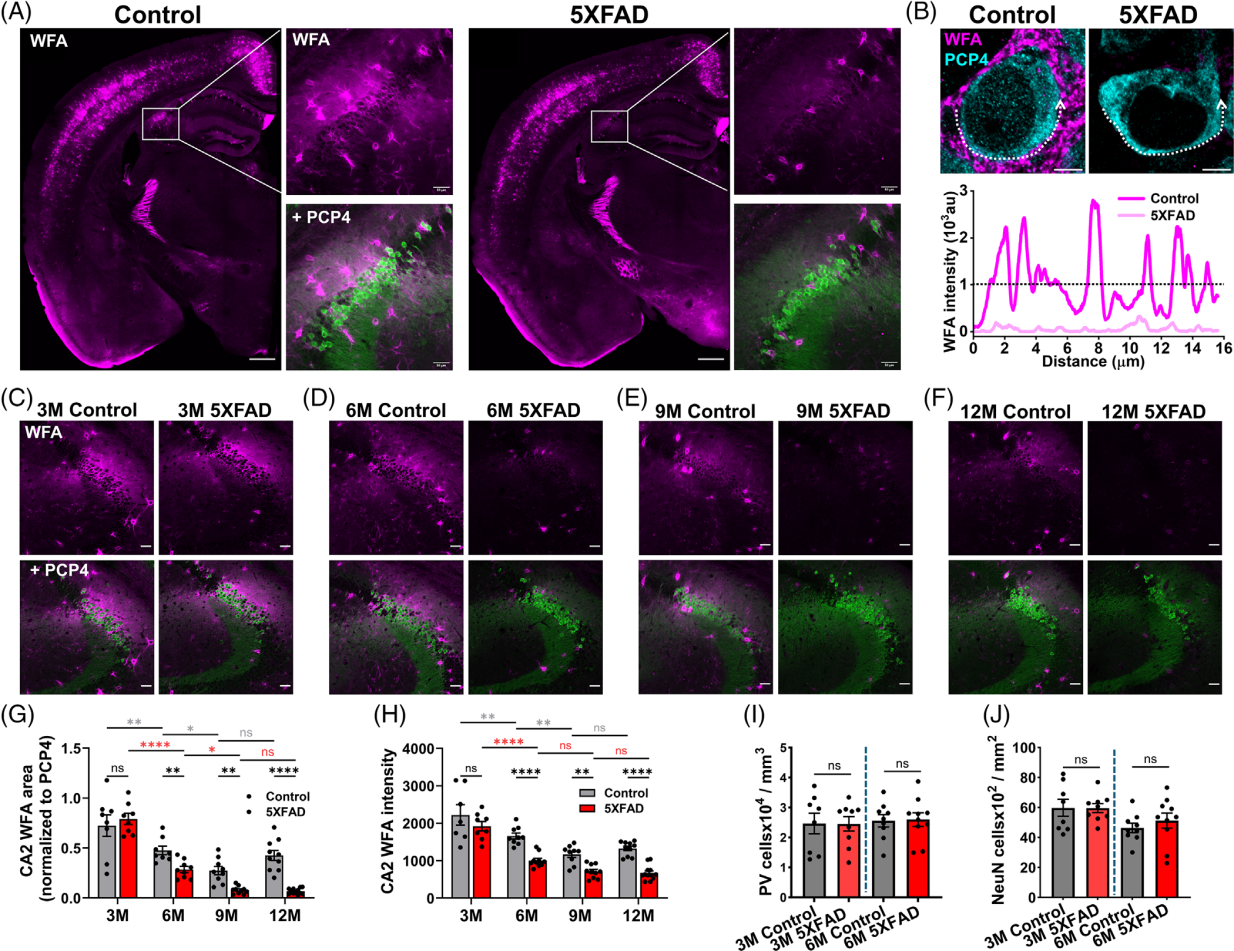
Progressive disruption of Hippocampal CA2 PNNs in 5XFAD mice. (A) Confocal micrographs showing immunohistochemical expression of WFA (magenta)‐labeled PNNs in coronal half‐brain sections from control and 5XFAD mice. Magnified areas in white rectangles highlight disruption of PNNs around hippocampal CA2 pyramidal neurons labeled with PCP4 (green). Scale 500 µm main images, 50 µm magnified images. (B) High magnification confocal micrographs of a single CA2 pyramidal neuron showing PNN disruption in 5XFAD and consequent loss of characteristic high‐intensity peaks and valley pattern in the line intensity profile (bottom). A line was drawn along the WFA signal in the PCP4 neuron periphery in control and 5XFAD group, showing many high‐intensity WFA peaks in control (dark magenta line) compared to the 5XFAD (light magenta line) group. Scale 2 µm. (C–F) Representative confocal micrographs of PNN (WFA‐magenta) immunofluorescence associated with CA2 pyramidal neurons (PCP4‐green) in 5XFAD mice at 3 (C), 6 (D), 9 (E), and 12 M (F) groups showing progressive PNN loss. Scale 50 µm. (G–H) Bar graphs of CA2 PNN area coverage (WFA/PCP4 area) (G), and CA2 PNN (WFA) intensity (H) in 5XFAD and their age‐matched control mice at 3, 6, 9, and 12 M showing significant decreases at and after 6 M in 5XFAD; *n* = 7–8 slices from five mice per group in 3 M, *n* = 9–10 brain slices from five mice per group in 6 M, *n* = 9–10 brain slices from five mice per group in 9 M, *n* = 10–12 brain slices from five mice per group in 12 M. (I–J) PV neuron density (I), and NeuN neuron density (J) in CA2 area at 3 and 6 M 5XFAD mice compared to their age‐matched controls showing no difference between any groups; *n* = 8–9 brain slices from five mice per group in 3 M, *n* = 9–10 brain slices from five mice per group in 6 M. Bar data in G–H indicate mean ± SEM and dots represent data points. Two‐way ANOVA, Tukey's multiple comparisons test in G–H; unpaired two‐tailed *t*‐test in I–J. *p < 0.05, **p < 0.01, ***p < 0.001,****p < 0.0001, and ns *p* > 0.05.

PNNs stabilize synapses^30^ which in turn govern memory and cognition,^31^ the brain functions that are impaired in AD. We sought to determine whether PNNs are altered in hippocampus, a key brain area associated with memory function, and subsequently, whether and how PNN alterations may contribute to memory dysfunction in AD. Using WFA as a pan PNN marker, we performed IHC and compared 6‐month (M) ‐old 5XFAD mice with age‐matched controls. Although subtle changes in PNN expression were visible in some brain regions (Figure S3), the most striking changes were seen in the hippocampal CA2 area where co‐labeling of WFA with CA2 neuron marker, PCP4, revealed a profound loss of PNNs around CA2 pyramidal neurons (Figure 1A,B). The inseparable intermingling of PNN around pyramidal and PV neurons in the CA2 prevented us from analyzing their PNNs separately, however we observed relatively intense PV neuron‐associated PNNs in 5XFAD mice (Figure 1A‐D). To generalize these findings of a selective loss of PNNs in CA2, we assessed two more widely used AD models namely J20 and 3XTg, which also showed a conspicuous loss of PNN in CA2 area (Figure S4A‐C).

Since AD is a progressive disease, we assessed the time course of PNN loss in the CA2 area of 5XFAD mice. Using a binary thresholding analysis method as described previously,^15^ we quantified the PNN expression by computing the WFA‐labeled area as well as WFA fluorescence intensity in the CA2 area in 3, 6, 9, and 12 M old 5XFAD and age‐matched control mice. At 3 M, CA2 PNNs were indistinguishable between 5XFAD and controls (Figure 1C,G), however, at 6 M, we observed a significant reduction in CA2 PNN area in the 5XFAD mice (Figure 1D,G). This decrease in the PNN coverage persisted in the 9 and 12 M old 5XFAD (Figure 1E,F,G). As a complementary analysis metric, we computed WFA intensity in the CA2 area which was similarly reduced significantly at 6‐12 M in 5XFAD mice (Figure 1H). Note that normal aging also presented with a reduction in PNN coverage, yet the loss of PNNs was significantly more pronounced in 5XFAD mice (Figure 1G,H).

Next, we assessed the expression of aggrecan, a chondroitin sulfate proteoglycan critically required to orchestrate the PNNs. Aggrecan expression in CA2 at 6 M remains unaltered in 5XFAD mice (Figure S5A,B), suggesting the loss of CS GAGs accounting for the disruption of WFA‐expressing PNNs. Considering the aggressive deposition of amyloid beta in the 5XFAD model, we sought to determine whether degradation of CA2 PNNs coincides with the deposition of amyloid plaque in the CA2 area. Surprisingly, despite a widespread hippocampal deposition of amyloid beta plaques (Figure S6A,B), the CA2 area remained largely devoid of amyloid beta plaques (Figure S6A,C), suggesting no spatial correlation between plaque pathology and PNN disruption in the CA2 area.

Neuronal cell death is a hallmark in AD pathology therefore it is plausible that CA2 PNN disruption may be due to the loss of CA2 neurons that express PNNs. To assess this possibility, we used NeuN as a pan‐specific neuronal marker and PV to identify PV interneurons, respectively, and assessed the neuronal density in the CA2 area defined by PCP4‐stained area. We did not find any change in either PV (Figure 1I) or NeuN cell density at 6 M of 5XFAD compared to age‐matched controls (Figure 1J). These findings suggest that PNN disruption specifically in the hippocampal CA2 area is an early and pronounced pathological change shared across multiple AD models and occurs prior to neuronal cell loss.

### 3.2 CA2 PNN disruption coincides with social memory deficits in 5XFAD mice model of AD

**FIGURE 2:**
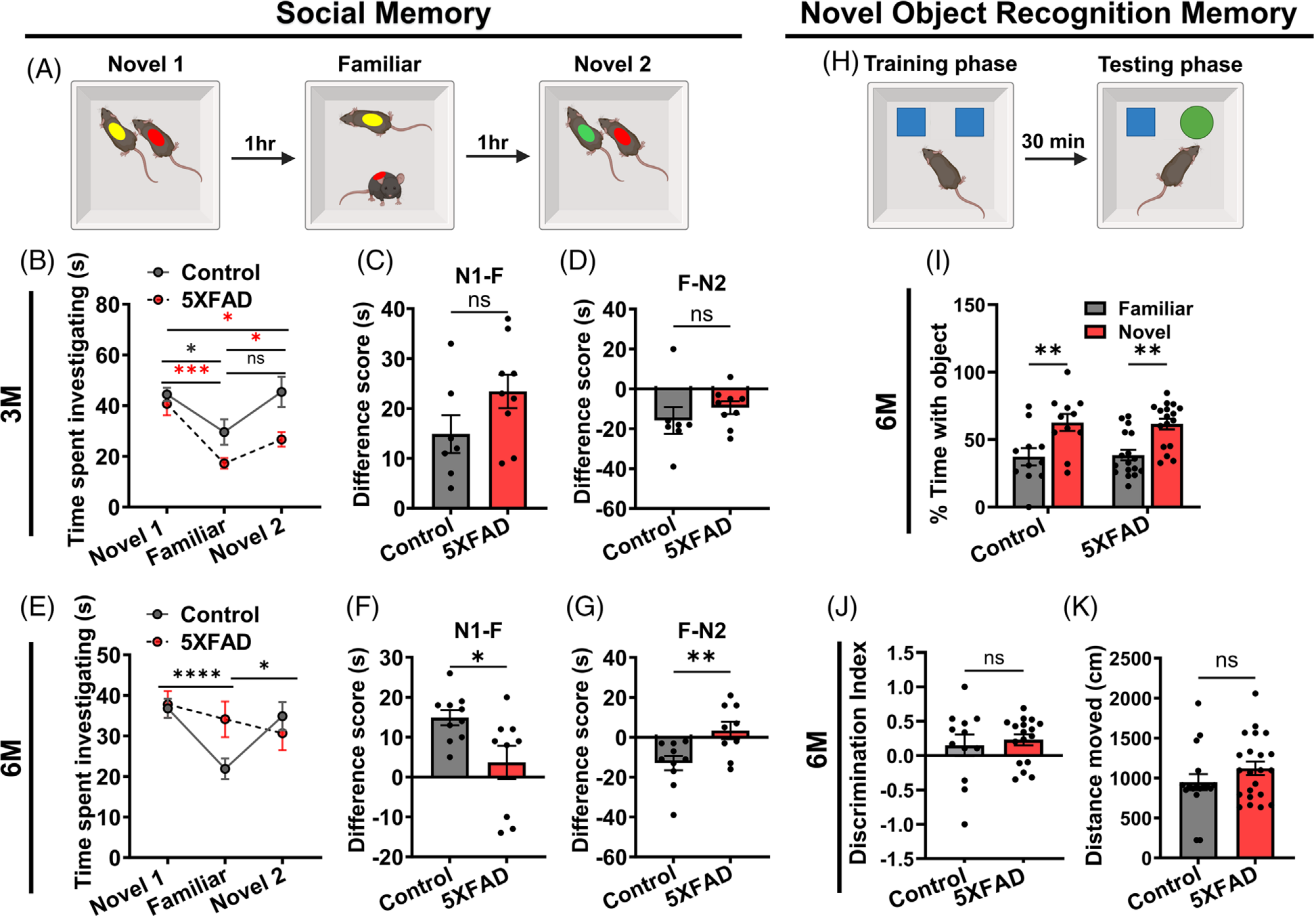
Impairment of social memory in 5XFAD mice at 6 M age. (A) Schematic of the direct interaction social memory test. The test mouse (red dot) interacts with Novel 1 mouse (yellow dot) who becomes Familiar (yellow dot) in the second trial. In the following trial, the test mouse (red dot) interacts with another novel (Novel 2) mouse (green dot). (B) Graph showing time spent investigating by 3 M old 5XFAD and age‐matched control mice with Novel 1, Familiar, and Novel 2 mice suggesting normal social memory (two‐way repeated measures ANOVA, Tukey's multiple comparisons test (*n* = 7) (control); 9 (5XFAD) mice; *p < 0.05, ***p < 0.001). (C–D) Bar graphs showing no significant change in difference scores, (C) Novel 1 – Familiar, and (D) Familiar − Novel 2, in 3 M 5XFAD mice compared to age‐matched control mice (*n* = 7) (control); 9 (5XFAD) mice in (C–D), unpaired two‐tailed *t*‐test. (E) Graph showing impaired social memory in 6 M 5XFAD mice compared to 6 M control mice (*n* = 10) (control); 9 (5XFAD) mice, two‐way repeated measures ANOVA, Tukey's multiple comparisons test; *p < 0.05, ****p < 0.0001. (F) Bar graph showing significant change in difference scores (Novel 1 − Familiar) in 6 M 5XFAD mice compared to 6 M control mice (*n* = 10) (control); 9 (5XFAD) mice; unpaired two‐tailed *t*‐test; *p < 0.05. (G) Bar graph showing significant change in difference scores (Familiar − Novel 2) in 6 M 5XFAD mice compared to 6 M control mice (*n* = 10) (control); 9 (5XFAD) mice; unpaired two‐tailed *t*‐test, ** *p* < 0.01. (H) Schematic of novel object recognition task. The test mouse is familiarized with two objects (blue), followed by replacing one object with a novel object (green) in the next trial. (I) Bar graph showing significantly higher time spent with the novel object compared to familiar by both 6 M control and 5XFAD (*n* = 11) (control); 17 (5XFAD) mice, two‐way ANOVA mixed‐effects Šídák's; **p < 0.01. (J–K) Bar graph showing no significant difference in discrimination index (J), and distance moved (K) by both 6 M control and 5XFAD (*n* = 11) (J), 17 (K) control; 17 (J), 22 (K) 5XFAD mice; unpaired two‐tailed *t*‐test.

The CA2 area of the hippocampus is recognized for its role in social recognition functions^19^, ^32^ and CA2 pyramidal neurons are known to regulate encoding, storage, and recall of social memory^20^. Since PNNs have been implicated in terminating synaptic plasticity^33^ and stabilizing memory traces,^31^ we hypothesized that PNN loss in CA2 may coincide with the onset of the social memory deficits in AD. To test this, we assessed social memory function in 3 and 6 M old 5XFAD mice representing the ages with intact and disintegrated PNNs, respectively. We used a well‐established paradigm,^22^ namely, a three‐trial direct social interaction test (Figure 2A) with a 1‐h interval between each trial. In the first trial, the test mouse was introduced to a novel mouse (Novel 1). In the second trial, the test mouse again encountered the previous Novel 1 mouse who now serves as the Familiar. In the third trial, the test mouse was introduced to a new novel mouse (Novel 2). During the second trial, the test mice with normal social memory spend less time interacting with the familiar mouse as they remember the interaction; however, in the third trial, they spend more time with a novel mouse (Novel 2).

We found that the 3 M old 5XFAD mice with intact PNNs behave identically to control mice, spending less time with the familiar mouse and more time with the novel mouse, with no significant difference in the difference scores between the groups (Figure 2 B‐D). By contrast, 6 M old 5XFAD mice, in which CA2 PNNs are disrupted showed impaired social memory. Specifically, unlike age‐matched controls, 5XFAD mice showed no difference in investigation times between the first novel mouse and the familiar mouse, nor between the familiar mouse and the second novel mouse (Figure 2E). By contrast, age‐matched control mice exhibited a predictable decrease in investigation time with the familiar mouse and an increase with the second novel mouse. The consistent interaction times across trials in 5XFAD mice suggest that their sociability was not affected by repeated interactions (Figure 2E). However, the lower difference scores in 5XFAD mice indicate a diminished ability to distinguish between familiar and novel mice (Figure 2F,G).

To determine if the PNN disruption correlates specifically with altered social memory or extended to other types of memories, we subjected mice to a novel object recognition (NOR) test in an open field arena (Figure 2H). NOR, a hippocampus‐dependent memory primarily involving the CA1 region, assesses the ability to distinguish between familiar and novel objects. During the training phase both control and 5XFAD mice did not show any preference toward right or left object (Figure S7). At 6 M, both control and 5XFAD mice demonstrated similar percentage of time spent with the familiar vs. novel object (Figure 2I), with no significant differences in the discrimination index between the groups (Figure 2J). These findings indicate that NOR remained intact in 6‐M‐old 5XFAD mice and that the memory deficits observed were specific to social memory.

Since 5XFAD mice exhibit motor impairments,^34^ we assessed whether change in exploratory locomotor activity could affect the social behavior. We measured the total travel distance in 6‐M‐old 5XFAD and control mice and found no significant difference between these groups (Figure 2K). These data confirmed that the observed social memory impairment in 5XFAD mice is not due to changes in general locomotory activity or exploratory behaviors.

### 3.3 CA2‐specific PNN disruption reversibly alters social memory

**FIGURE 3:**
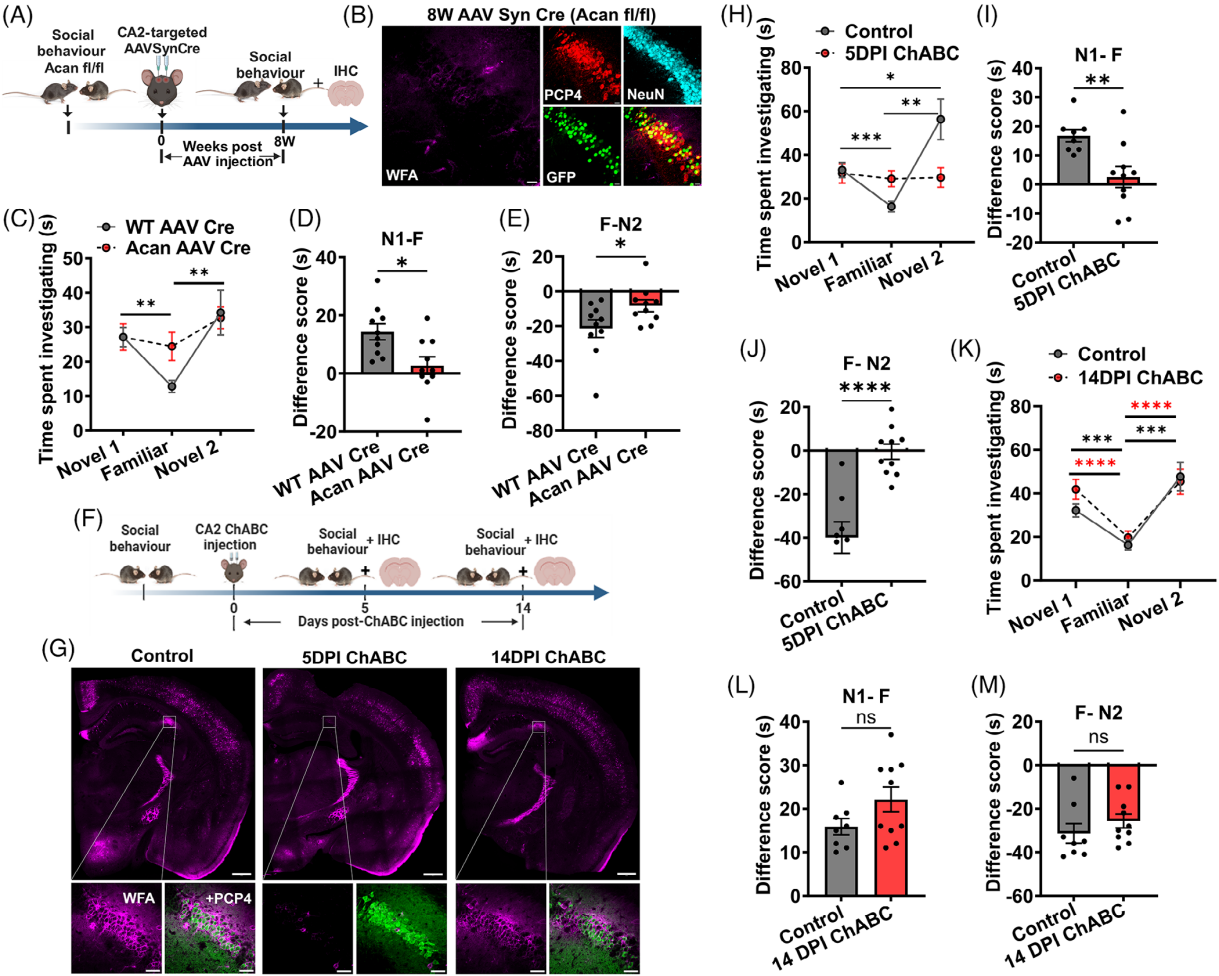
Genetic and enzymatic CA2 PNN disruption impairs social memory. (A) Schematic of social memory assessment before and after genetic deletion of CA2 PNNs. We tested social memory in *Acan^fl/fl^* and WT mice followed by CA2 targeted injection of AAVSynCre and after 8 weeks again measured social memory and performed IHC analysis. (B) Confocal micrograph shows PNN (WFA) deletion in AAV SynCre (GFP)‐ mediated Acan KO in CA2 neurons (PCP4, NeuN) in *Acan^fl/fl^* mice. Scale 10 µm. (C) Graph showing impaired social memory in *Acan^fl/fl^* mice injected with AAV SynCre (Acan AAV Cre) compared to the WT mice injected with AAV SynCre (WT AAV Cre), *n* = 10 mice in each group, two‐way ANOVA, Tukey's multiple comparisons test; **p < 0.01. (D–E) Bar graph showing a significant change in difference scores in (D) Novel 1 − Familiar, and (E) Familiar – Novel 2, in CA2 PNN deleted mice (Acan AAV Cre); *n* = 10 mice per group in (D) and (E), unpaired two‐tailed *t*‐test, *p < 0.05. (F) Schematic of social memory assessment before and after enzymatic PNN disruption. After testing social memory in WT mice, CA2‐targeted ChABC or PNase injections were done. Social memory was again assessed after 5‐ or 14‐DPI and brains were collected for IHC. (G) Confocal micrographs of PNN (WFA) immunofluorescence in CA2 neurons (PCP4) showing PNN disruption at 5DPI and homeostatic regrowth by 14 DPI in ChABC‐injected mice. Scale 500 µm main images, 50 µm magnified images. (H) Graph showing impaired social memory in ChABC‐injected mice at 5 DPI (5DPI ChABC) whereas PNase‐injected mice (control) show normal social memory (*n* = 8) (control) 10 (5DPI ChABC) mice; two‐way ANOVA, Tukey's multiple comparisons test; *p < 0.05, **p < 0.01, ***p < 0.001. (I–J) Bar graphs showing significant change in difference scores (I) Novel 1 − Familiar, and (J) Familiar – Novel 2, in 5 DPI ChABC mice compared to control mice (*n* = 8) (control), 10 (5 DPI ChABC) in both (I) and (J); unpaired two‐tailed *t*‐test; **p < 0.01, ****p < 0.0001. (K) Graph showing reinstatement of social memory in ChABC‐injected mice by 14 DPI (*n* = 8) (control), 10 (14 DPI ChABC) mice; two‐way ANOVA, Tukey's multiple comparisons test; ***p < 0.001, ****p < 0.0001. (L–M) Bar graphs showing no change in difference scores (L) Novel 1 − Familiar, and (J) Familiar – Novel 2, in 14DPI ChABC mice compared to control mice (*n* = 8) (control), 10 (14 DPI ChABC) in both (L) and (M); unpaired two‐tailed *t*‐test.

To examine a causal relation between our observed disrupted CA2 PNNs and deficits in social memory in 5XFAD mice, we assessed social memory before and after experimental PNN elimination in control mice. To this end, we deleted Acan, an indispensable CSPG constituent of the PNN, through CA2‐targeted bilateral injection of AAV SynCre into *Acan ^fl/fl^* mice. We began by confirming the normal social behavior of *Acan ^fl/fl^* and WT mice (Figure S8) followed by bilaterally injecting AAV SynCre targeting the CA2 area and assessing social behavior after 8 weeks. Subsequently, we performed immunohistochemical analysis to confirm CA2 PNN deletion (Figure 3A). Corroborating our recent studies in cerebral cortex,^15^ the vast majority of WFA‐expressing PNNs were eliminated after 8 weeks of AAVCre injection in *Acan ^fl/fl^* mice (Figure 3A,B). However, a minor population of CA2 neurons showed modest reduction in Acan and WFA fluorescence intensities (Figure S9A‐C), suggesting a partial knockout of PNNs. WT mice injected with AAV SynCre displayed unaltered PNNs (Figure S9D) as well as normal social memory, spending less time with the familiar mouse and more with the novel mouse after a 1‐h delay. By contrast, *Acan ^fl/fl^* mice injected with AAV SynCre showed impaired social memory as evidenced by no difference in interaction times between familiar and novel mice (Figure 3C). The difference score between the two groups was significantly different (Figure 3D,E), indicating that deletion of CA2 PNNs impairs social memory. Occasionally, we observed a minimal AAV spread to the somatosensory area and CA2 flanking areas (Figure S9). These areas have a lower numerical density of PNN, as only PV neurons have PNNs compared to the CA2 area, where PNNs are around all neurons.

We next asked whether reinstating CA2 PNNs can reverse the social memory deficits induced by the PNN disruption. We took advantage of the ability to transiently and reversibly degrade PNNs with ChABC,^23^ a bacterial‐derived enzyme that has been utilized to degrade PNNs in numerous studies.^22^, ^23^ As shown in Figure 3F,G, this approach eliminates PNNs by 5 days post‐injection (DPI), yet PNNs regrow by 14 DPI as also shown in a recent study.^22^ Similar to our Acan KO studies (Figure 3A‐E), we first confirmed normal social memory in WT mice, subsequently bilaterally injected ChABC targeting the CA2 area followed by testing social memory at 5 DPI and 14 DPI (Figure 3F). Age‐matched control mice were injected with PNase, an enzyme that does not degrade PNNs and has been used in comparable studies.^22^ On completion of social behavior experiments, we performed immunohistochemical analysis to confirm PNN disruption or recovery using WFA staining (Figure 3F,G).

At 5 DPI, ChABC‐treated mice showed disrupted CA2 PNNs (Figure 3G). ChABC‐treated mice did not show an increase in interaction with familiar and novel mice indicating no change in sociability (Figure 3H). In addition, the difference score of the ChABC‐treated group was significantly lower than the control at 5DPI (Figure 3I, J). By 14 DPI, PNNs reappeared in the CA2 area (Figure 3G). Interestingly, the social memory was also reinstated in ChABC‐treated mice by 14DPI, as they exhibited normal social interaction behavior with familiar and novel mice, similar to PNase‐treated control mice (Figure 3K). No difference was observed in the difference scores between the groups (Figure 3L,M). To rule out the non‐specific effect of protease and other unknown peptides reported in sigma ChABC,^35^, ^36^ we compared the effect of heat‐inactivated ChABC control with the PNase injected controls. Both conditions showed no effect on PNNs (Figure S1A‐E).

In conjunction with the AAVSynCre‐*Acan ^fl/fl^* studies, these results demonstrate that the elimination of PNNs is sufficient to impair social memory, and restoring PNNs can reverse social memory deficits. Therefore, it is likely that the pathological CA2 PNN disruption is causally associated with altered social memory behavior in the 5XFAD mice.

### 3.4 Disequilibrium of ECM and PNN synthesis and remodeling in CA2 area in AD mice

To gain insight into possible mechanisms underlying the PNN disruption in CA2 of 5XFAD mice, we began with an unbiased analysis of gene expression in the hippocampal CA2 area of these mice. We used 3 and 7 M old 5XFAD mice as comparative groups with confirmed social memory deficits at 7 M. The 3 M age represents the commencing phase of PNN disruption with still intact social memory while the 7 M group represents an established phase when PNNs are chronically altered with an underlying social memory deficit.

**FIGURE 4:**
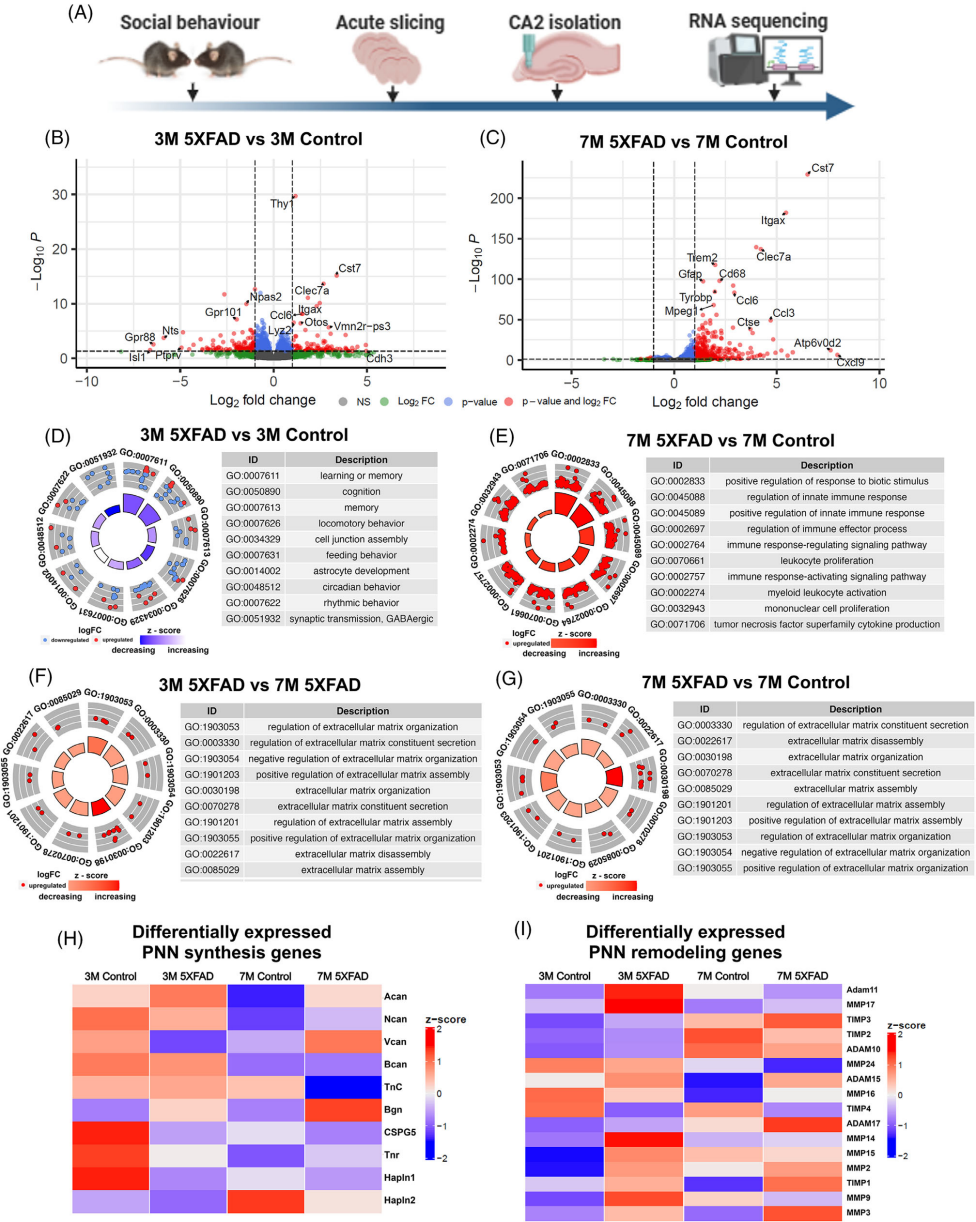
Transcriptomic analysis of age‐dependent CA2 area gene expression changes in 5XFAD mice. (A) Schematic of experimental design. We tested social memory in 3 and 7 M old control and 5XFAD mice followed by acute brain slicing and isolation of the CA2 area using a tissue puncher. CA2 area tissues were frozen fixed and processed for bulk RNA‐sequencing. (B) Volcano plot showing DEGs in the CA2 area in 3 M 5XFAD and age‐matched control mice. Genes with an adjusted *p*‐value < 0.05 are shown in blue, genes with log_2_ fold‐change > 1.00 in green, and genes meeting both criteria in red. Gray dots represent genes that do not meet either criterion. (C) Volcano plot showing DEGs in the CA2 area in 7 M 5XFAD and age‐matched control mice. Color coding is consistent with that in (B). (D–E) GO term circle plot highlighting the top 10 GO terms, sorted by adjusted *p*‐value in (D) 3 M, and (E) 7 M, 5XFAD versus age‐matched control mice. Z‐scores indicate the directionality of regulation, with red reflecting upregulated genes and blue reflecting downregulated genes. (F, G) GO term circle plot of the top 10 GO terms related to PNNs and ECM, ranked by adjusted *p*‐value. Z‐scores indicate the directionality of regulation, with red reflecting upregulated genes. (H–I) Heatmaps showing the expression levels of key genes involved in, (H) PNN synthesis, and (I) PNN degradation across all samples and experimental conditions. Expression values were normalized by z‐score transformation.

We isolated the CA2 area by punching acute hippocampal slices with a tissue puncher and performed bulk RNA‐seq (Figure 4A). In 3 M old 5XFAD mice compared to control, we found 98 upregulated genes and 133 downregulated genes (padj < 0.05 and log2FC < −1.00) (Figure 4B). In 7 M old 5XFAD mice compared to control, there were 314 upregulated genes and 6 downregulated genes (padj < 0.05 and log2FC > 1.00) (Figure 4C). When looking at the top 10 most significant Gene ontology (GO) Terms for 3 M (Figure 4D) and 7 M groups (Figure 4E), we found many pathways associated with learning, memory, and cognition are downregulated in 3 M old 5XFAD mice compared to their age‐matched controls (Figure 4D). Whereas many pathways related to immune response were upregulated in 7 M old 5XFAD mice compared to their age‐matched controls (Figure 4E). GO term of the 3 M 5XFAD versus 7 M 5XFAD and 7 M 5XFAD versus 7 M control revealed changes in genes associated with the ECM organization, assembly, disassembly, and secretion (Figure 4F,G). Consistent with intact memory, 3 M 5XFAD versus 3 M control did not show any change in these genes. Overall, we observed an upregulation of pathways associated with immune activation, along with alterations in memory and ECM‐related pathways.

Next, we narrowed down our analysis to the genes associated with PNN synthesis (Figure 4H) and remodeling processes (Figure 4I). The 3 M old 5XFAD mice compared to their controls showed unchanged expression of many genes involved in PNN synthesis; however, a few genes including Vcan, CSPG5, Tnr, and Hapln1 were downregulated (Figure 4H). Similarly, we found an increase in Vcan and Bgn components, while other genes remained mostly unaltered in 7 M old 5XFAD mice compared to their controls (Figure 4H).

In contrast to the broadly stable expression of PNN synthesis‐related genes, the PNN remodeling genes were largely upregulated. 3 M old 5XFAD compared to their age‐matched controls shows an increase in MMP 2, 3, 9, 14, 15, 17, TIMP 1, 2, 3, and ADAM 10, 11, 15, 17. The 7 M old 5XFAD compared to their age‐matched controls shows an increase in MMP 2, 3, 16, TIMP1, 3, and ADAM 15, 17. The common genes upregulated at both 3 and 7 M 5XFAD compared to their age‐matched controls were MMP2, MMP3, and TIMP1 (Figure 4I), whereas 7 M 5XFAD compared to 3 M 5XFAD mice show an increase in MMP3, TIMP1, 2, 3, Adam 10, 17 (Figure 4I). Overall, our bulk RNA‐seq data from the CA2 area reveals an imbalance in the expression of PNN synthesis and remodeling‐related genes in 5XFAD mice.

### 3.5 Blocking MMP activity prevents PNN disruption and retains social memory in AD mice

The elevated MMP transcript levels (Figure 4I) closely coincide with the disrupted PNNs (Figure 1G,H) and altered social memory (Figure 2E‐G) in AD mice, therefore we investigated whether inhibiting the proteolytic function of MMPs using a broad‐spectrum inhibitor could prevent PNN disruption and in turn preserve social memory in the AD mice. Given the upregulation of many MMPs in our CA2 bulk RNA sequencing analysis, we selected GM6001 or Ilomastat for its broad inhibitory activity against multiple MMPs, including MMP‐1, MMP‐2, MMP‐3, MMP‐7, MMP‐8, MMP‐9, MMP‐12, MMP‐14, and MMP‐26.

**FIGURE 5:**
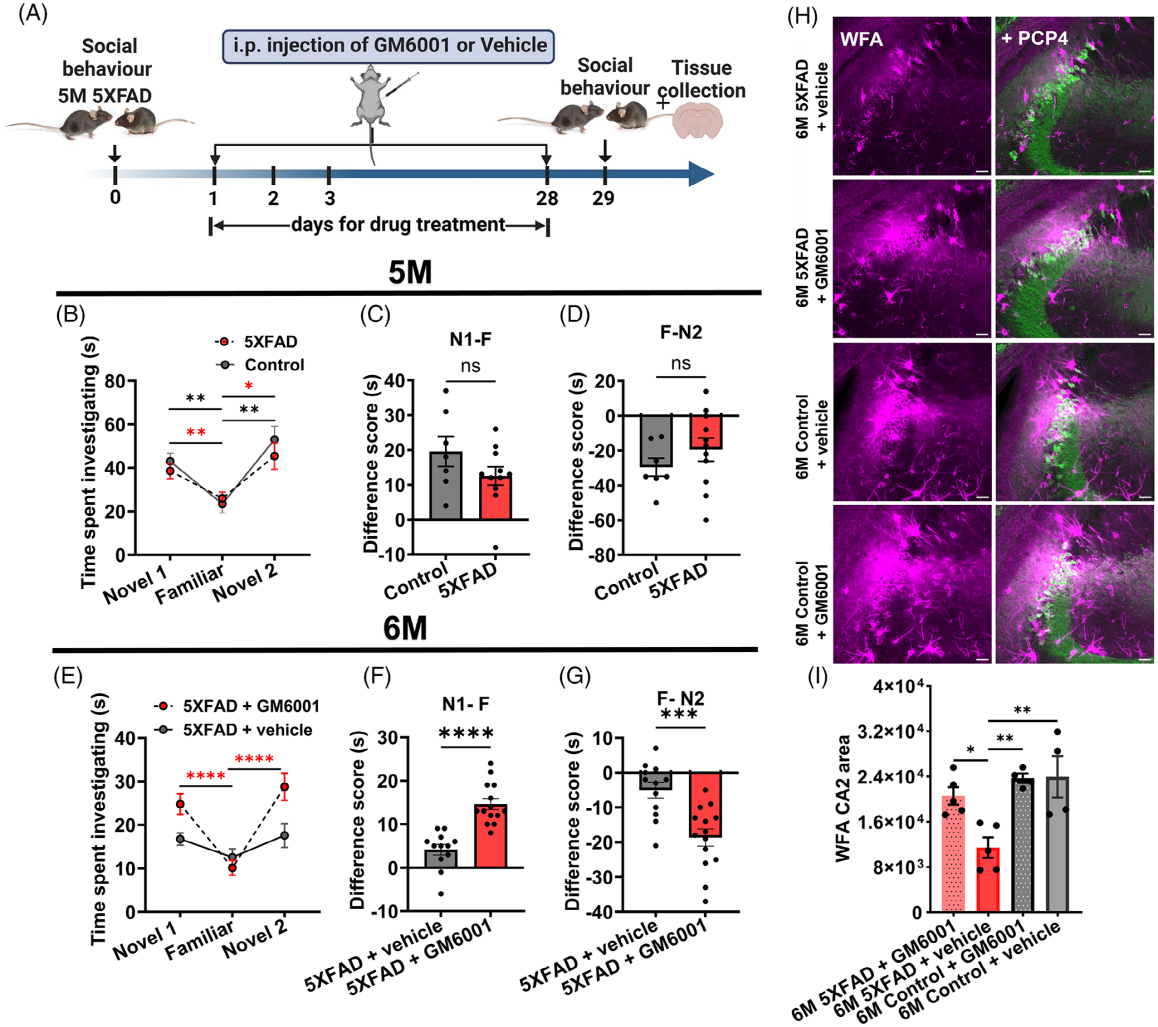
The MMP blocker GM6001 delays social memory deficits and inhibits PNN degradation in 5XFAD mice. (A) Schematic of experimental design. We assessed social memory in 5 M 5XFAD and age‐matched control mice followed by i.p. GM6001 or vehicle injections for 1 month. Mice were again assessed for social memory after the injections and brains were collected for IHC. (B) Graph showing normal social memory in 5 M 5XFAD and age‐matched control mice (*n* = 7) (control), 11 (5XFAD) mice; two‐way ANOVA, Tukey's multiple comparisons test; *p < 0.05, **p < 0.01. (C–D) Bar graphs showing no significant change in difference scores, (C) Novel 1 – Familiar, and (D) Familiar − Novel 2, in 5 M 5XFAD mice compared to age‐matched control mice (*n* = 7) (control); 11 (5XFAD) mice in (C–D), unpaired two‐tailed *t*‐test. (E) Graph showing normal social memory in a month‐long GM6001‐injected 6 M 5XFAD mice whereas vehicle‐injected 6 M 5XFAD mice show disrupted social memory compared to the age‐matched control mice (*n* = 14) (5XFAD + GM6001), 12 (5XFAD + vehicle) mice; two‐way ANOVA, Tukey's multiple comparisons test; *p < 0.05, ****p < 0.0001. (F–G) Bar graphs showing significant difference in difference scores (F) Novel 1 – Familiar, and (G) Familiar − Novel 2, in GM6001‐injected 6 M 5XFAD in 6 M compared to vehicle‐injected 6 M 5XFAD mice (*n* = 14) (5XFAD + GM6001), 12 (5XFAD + vehicle) mice in (F–G); unpaired two‐tailed *t*‐test; ***p < 0.001, ****p < 0.0001. (H) Representative confocal micrographs of PNN (WFA) immunofluorescence in CA2 area (PCP‐4) from 6 M 5XFAD mice injected with vehicle (top), or GM6001 (2nd from top), and 6 M Control (5XFAD‐) mice injected with vehicle (3rd from top) or GM6001 (bottom). Scale 50 µm. (I) Bar graphs showing significantly higher CA2 PNN expression in GM6001‐treated mice compared to vehicle‐treated 5XFAD mice whereas age‐matched GM6001‐treated or vehicle‐treated controls exhibited PNN expression comparable to 6 M GM6001‐treated 5XFAD mice (one‐way ANOVA, Tukey's multiple comparisons test; *n* = 4–5 mice per group; *p < 0.05, **p < 0.01).

To establish the GM6001 treatment timeline, we further narrowed down the time point of onset of PNN disruption and consequent social memory deficits. Upon examining ages between 3 and 6 M in 5XFAD mice, we found that PNNs and social memory were normal until 5 M of age; however, by 6 M both are impaired (Figure 5B–D, 2E–G). Therefore, we initiated GM6001 treatment at the age of 5 M (Figure 5A) when these mice exhibited normal social memory behavior with no significant difference in the difference score between 5 M old 5XFAD and age‐matched controls (Figure 5B–D).

We injected 100 mg/kg/day (i.p.) GM6001, which was previously shown to be effective in blocking MMP activity in glioma‐associated epilepsy^29^ and stroke.^37^ Our behavior study included two groups: 5XFAD mice treated with GM6001 or vehicle. Following 1 M of treatment when mice turned 6 M old, we assessed social behavior in 5XFAD mice treated with vehicle and GM6001. 5XFAD mice treated with vehicle showed altered social memory behavior; however, the GM6001‐treated 5XFAD mice exhibited normal social behavior, with a significant difference in the difference score compared to vehicle‐treated 5XFAD mice (Figure 5E–G). Besides normal social behavior, the IHC analysis of GM6001‐treated mice brains exhibited significantly higher CA2 PNN expression compared to vehicle‐treated 5XFAD mice (Figure 5H,I). We also included age‐matched controls treated with GM6001 or vehicle for IHC analysis. Age‐matched controls treated with GM6001 or vehicle exhibited PNN expression comparable to GM6001‐treated 5XFAD mice, indicating GM6001 had no significant effect on control mice (Figure 5H,I). In addition, the control mice treated with GM6001 and vehicle showed comparable PNN expression (Figure 5H,I).

These findings suggest that blocking MMP activity before the onset of PNN disruption in 5XFAD mice can prevent PNN degradation and consequently avert the loss of social memory.

## 4 DISCUSSION

Recent studies provide compelling evidence of a progressive loss of PNNs in animal models^8^, ^18^ and human AD brains.^4^, ^8^, ^38^ Nevertheless, the causes and consequences of PNN disruption in AD remain poorly understood. Interestingly, nearly all PNN‐related studies in AD focus on the changes in the PNNs that are typically expressed around the PV‐expressing GABAergic neurons. By contrast, in the present study, we report a pronounced disruption of PNNs expressed on excitatory glutamatergic pyramidal neurons in the hippocampal CA2 area. We demonstrate that loss of CA2 PNNs in AD brains causes social memory dysfunction which can be prevented by inhibiting the proteolysis of PNNs using a broad‐spectrum inhibitor of MMPs.

We observed a profound disruption of PNNs in the CA2 area of the hippocampus in 6 M old 5XFAD mice. The loss of PNNs appears to be chronic and irreversible as older ages including 9 and 12 M old 5XFAD mice continue to exhibit PNN loss. These PNNs are unique as they encapsulate the CA2 excitatory pyramidal neurons intermingled with a small population of classic PNN‐expressing PV neurons. The pronounced loss of PNNs appears to be selective on CA2 pyramidal neurons while the typical PNNs on the PV neurons in the hippocampus and other brain regions show only subtle changes at 6 M (Figure S3). We also observed CA2 PNN disruption in J20 as well as in 3XTg models suggesting that CA2 PNN disruption may be a core pathology across multiple models of AD. To the best of our knowledge, these are novel findings as none of the previous studies have reported a specific loss of PNNs associated with CA2 pyramidal neurons in these AD models. Numerous studies^39^ have reported changes in PNNs and ECM constituents in the human AD brains however the fate of CA2 PNNs remained largely unexplored, plausibly due to a low expression of PNN markers in human CA2.

The progressive nature of AD requires a well‐defined timeline of pathological changes to be considered for any treatment approach. To this end, we established the time course of the PNN disruption in the 5XFAD model of AD which in conjunction with the recent literature^8^ suggests a precocious degradation of CA2 PNNs potentially indicating a high susceptibility of CA2 neurons. This aligns well with the fact that PNNs are vulnerable to oxidative stress,^40^ which in this case may be associated with the inflammation associated with AD.^41^ Interestingly, the disruption of PNNs was not due to the loss of pyramidal neurons as is commonly seen in AD brains^42^ and animal models.^43^ Instead, we found altered expression of numerous genes associated with PNN synthesis and remodeling pathways in the CA2 neurons suggesting ECM‐ and PNN‐related changes as core pathological features with an early onset in AD brains.

The conspicuous loss of CA2 PNNs in 5XFAD prompted us to explore its impact on the AD etiology. We found that CA2 PNN disruption in 5XFAD mice also presented with a loss of social recognition memory while object recognition memory remained unaltered. Our AAVSynCre–*Acan ^fl/fl^* studies convincingly establish a causal role of CA2 PNN disruption on social memory deficits as control mice with initially normal social memory and CA2 PNN develop social memory deficits upon deletion of their CA2 PNNs. Notably, the genetic approach specifically eliminates PNNs, unlike the enzymatic degradation that indiscriminately cleaves the proteoglycans in PNNs and interstitial space. However, enzymatic PNN digestion is advantageous to answer whether social memory dysfunction can be rescued on homeostatic PNN regrowth. Accordingly, we show a temporary disruption of social memory on enzymatic digestion of CA2 PNNs which reappears on the homeostatic reformation of the PNNs. Although the enzymatic digestion spread to nearby cortical and hippocampal areas, it is not likely that this influenced social memory as PNNs in these areas are not implicated in social recognition memory. These findings align well with the emerging literature suggesting a pivotal role of CA2 neurons and their PNNs in the social recognition function.^19^, ^44^ In particular, abnormal CA2 PNNs impair the social memory in an inbred mouse strain of social dysfunction, and maintaining a physiological level of PNNs rescues social memory.^22^ A recent study correlates the loss of PV‐associated PNNs in CA2 with the social memory deficits in the Tg2576 model of AD,^18^ while another study^44^ suggests that pyramidal neuron‐specific PNN knockout is sufficient to induce social memory deficits. Although our study does not rule out the involvement of PNNs on CA2 PV neurons, in conjunction with Alexander et al.,^44^ our findings on CA2‐specific genetic KO of PNNs in the adult brain support the idea that CA2 pyramidal neuron‐specific PNNs critically determine the social memory. One interesting aspect of our study is the loss of WFA‐labelled PNNs while aggrecan‐labelled PNNs remained unaltered. This suggests that the loss of PNNs may be due to the disruption of GAGs, but not due to the aggrean core protein. Accordingly, our approach of aggrecan knockout to eliminate PNNs has an underlying limitation that it deletes the aggrecan core protein as well as GAGs. However, since aggrecan remains unaltered in AD mice with impaired social recognition memory, it suggests a dispensable role of aggrecan core protein in social recognition memory.

The molecular mechanisms whereby PNN alterations impact brain functions including learning and memory can be diverse and are still under active investigation. PNNs have long been known to restrict synaptic plasticity thereby stabilizing synapses and preventing any unwanted change in the memory trace. At the molecular level, this synaptic stability can be attributed to the PNN which maintains a spatiotemporally stable expression and functional activity of ion channels and neurotransmitter receptors at synapses.^45^ Our recent study also suggests a pivotal role of PNNs in preventing ion and neurotransmitter spillage at synapses, thereby preventing activation of extrasynaptic receptors.^15^ In the specific case of CA2 pyramidal neuron‐associated PNNs, it has been shown that CA2 PNN disruption enables the induction of synaptic potentiation and increases excitatory synaptic activity in CA2 neurons.^33^ More recent studies suggest that CA2 PNN stabilizes synaptic inputs from the supramammillary nucleus onto CA2 pyramidal neurons.^44^ Based on these studies, we speculate that AD‐associated PNN loss not only destabilizes the synaptic inputs on CA2 neurons but also alters synaptic ion and neurotransmitter homeostasis.^15^

In addition to demonstrating the consequences of CA2 PNN loss in AD, our study also provides a mechanistic insight into the cause of CA2 PNN disruption and treatment strategies to prevent PNN disruption thereby social memory deficits. PNNs are multimolecular aggregates therefore their homeostatic maintenance is orchestrated by a set of diverse molecules. Similarly, PNN dismantling is effectuated by a large family of metalloproteinases whose expression and activity are tightly controlled at multiple levels.^15^, ^46^

A delicate balance between homeostatic replacement and remodeling of PNNs maintains their physiological expression. Our CA2 area‐specific RNA‐Seq data of different ages show a progressive establishment of a disequilibrium between PNN synthesis and dismantling mechanisms which, with the disease progression disrupts PNNs and causes social memory dysfunction. We postulate that, at the early or presymptomatic phase of AD (3 M age in 5XFAD), a modest reduction in the PNN synthesis machinery in tandem with a multifold increase in the PNN remodeling metalloproteinases, particularly MMP14, triggers the ECM disequilibrium. However, this disequilibrium is still not strong enough to significantly alter PNNs due to their continuous replacement. PNN disruption may also be further delayed by a compensatory overexpression^47^ of TIMPs, especially TIMP1, that inhibit MMP activity. Therefore, initially, PNNs and social memory are insignificantly affected. However, as the disease progresses with the chronic upregulation of MMPs and ADAMTs in the AD brain, the PNN dismantling eventually outweighs its homeostatic replacement and causes social memory deficits. Since chronic overexpression of MMPs drives the disequilibrium leading to disruption of the PNNs, thereby causing social memory deficits, we postulated that blocking MMP activity at an early stage of AD can be a promising strategy to prevent PNN disruption and thereby deficits in social memory. This is indeed supported by our studies on 5XFAD mice treated with a broad‐spectrum MMP inhibitor GM6001, which preserves PNNs and retains social memory, suggesting this class of drugs as a potential treatment for AD.

In summary, our study uncovers a novel, cell‐type‐specific loss of PNNs enveloping excitatory pyramidal neurons in the CA2 area of the hippocampus in multiple mouse models of AD. Unlike the modest alterations in PV neuron‐associated PNNs, CA2 pyramidal neuron PNNs exhibit a striking and progressive disruption that precedes overt neurodegeneration and correlates with deficits in social recognition memory. Mechanistically, RNA‐seq analysis reveals that this loss is driven by a disease‐stage‐specific imbalance between PNN synthesis and degradation pathways, characterized by downregulation of PNNs and concurrent upregulation of MMPs and ADAMTS family proteases, implicating these specialized ECM structures as essential regulators of social memory encoding in the hippocampus. Importantly, pharmacological inhibition of MMPs using GM6001 restores PNN integrity and rescues social memory deficits, positioning MMP‐mediated ECM remodeling as a mechanistic driver of cognitive dysfunction in AD.

These findings define CA2 PNNs as key substrates of circuit vulnerability in AD and underscore the importance of ECM remodeling dynamics in disease progression. Future work should aim to delineate the upstream regulators that control MMP and ADAMTS expression specifically in CA2 pyramidal neurons, including contributions from local inflammatory signaling, oxidative stress, and activity‐dependent pathways. Understanding how disrupted PNN integrity alters input–output relationships in CA2, particularly those involving the supramammillary and entorhinal cortex pathways could reveal circuit‐level mechanisms underlying social memory loss. Since PNNs regulate neuronal excitability, thereby excitation‐inhibition balance to prevent seizures, future studies can examine the role of CA2 PNN loss in AD‐associated seizures. Finally, targeted modulation of ECM remodeling enzymes or stabilization of PNN components represents a promising therapeutic strategy warranting further preclinical and translational exploration.

## AUTHOR CONTRIBUTIONS

L.C. conceived the idea, designed, performed, analyzed, and interpreted IHC, microscopy, behavior, drug studies, and RNAseq experiments, and wrote the original manuscript draft. J.L. and E.F. analyzed RNAseq data. C.P. and S.H. assisted with IHC and behavior. I.K. assisted with behavior. S.J. assisted with drug studies. M.E. assisted with drug studies and RNA isolation. E.C. assisted with behavior analysis. M.O. assisted with RNAseq data interpretation. B.P.T. conceived the idea and assisted with experimental design, data interpretation, manuscript writing, and editing. H.S. conceived the idea and carried out project supervision, data interpretation, manuscript writing, editing, and funding acquisition.

## CONFLICT OF INTEREST STATEMENT

All authors declare no conflict of interest. Author disclosures are available in the Supporting Information.

## CONSENT STATEMENT

No human subjects were involved in this study.

## Supporting information



Supporting Information

Supporting Information

Supporting Information

Supporting Information

Supporting Information

Supporting Information

## Data Availability

Data will be provided upon reasonable request to the corresponding authors.
