# Supplementary material for: Degradation of perineuronal nets in hippocampal CA2 explains the loss of social cognition memory in Alzheimer's disease
Source: Alzheimers Dement. 2025 Oct 22;21(10):e70813. doi: 10.1002/alz.70813 (PMC12541281; doi:10.1002/alz.70813)
Supplement: Supplementary file 5 — Supporting Information [file ALZ-21-e70813-s006.docx]

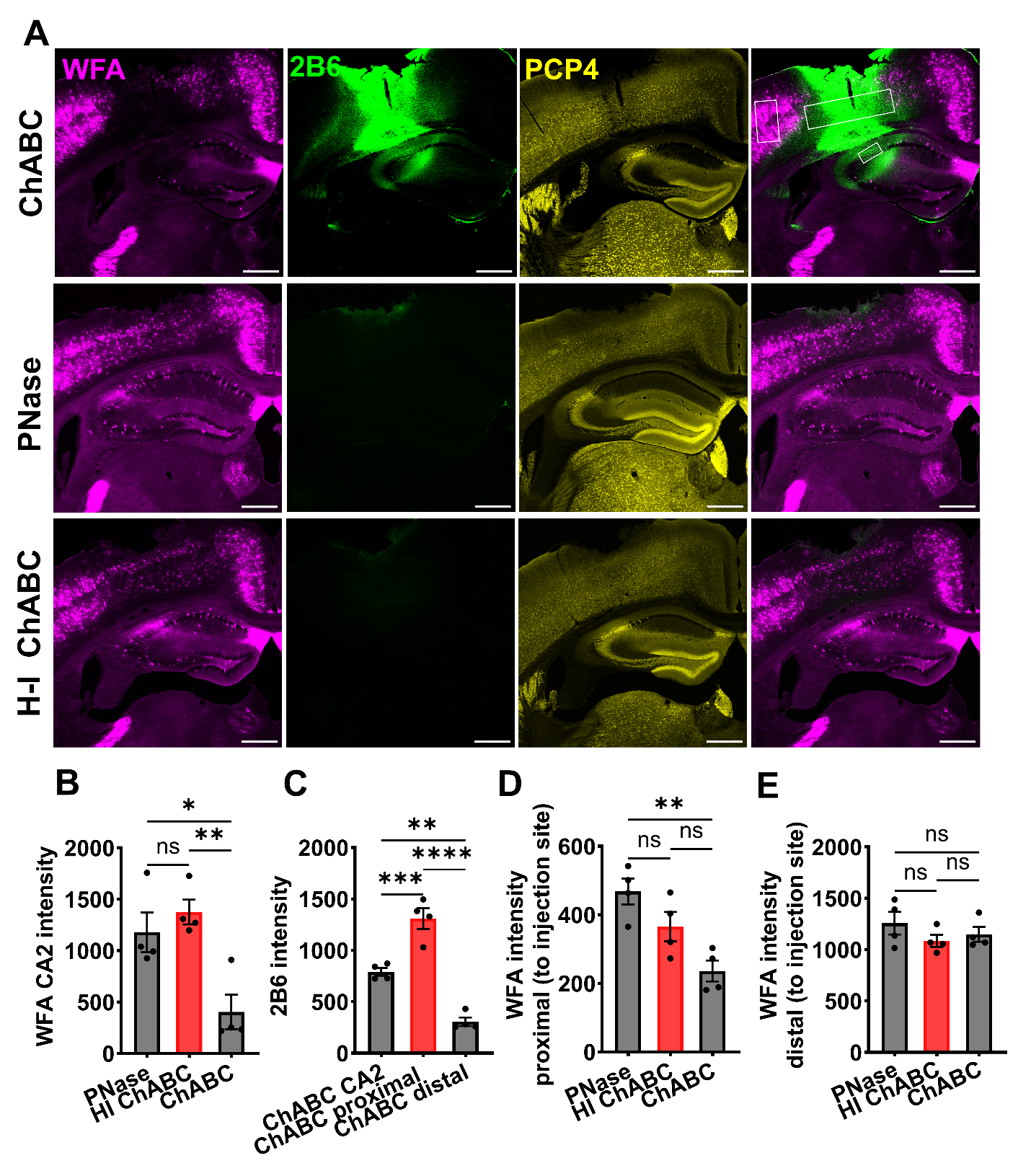
**Figure S1. PNN disruption using ChABC injection *in-vivo* mouse brains**

(A) Representative confocal micrographs showing immunohistochemical expression of PNN (WFA), CS stub (2B6), and CA2 neuron marker (PCP4) in mice brains injected with ChABC, PNase, and Heat-inactivated (H-I) ChABC. Areas in the smallest, medium, and largest white rectangles mark the targeted injection site (CA2), distal to the injection site, and proximal to the injection site, respectively. Scale = 500µm. Note that the images in ChABC and PNase groups are from different mice, however, from the same cohort that was used for acquiring images in Fig 3G.

(B) Bar graphs showing WFA fluorescence intensity in CA2 area of PNase, heat-inactivated ChABC (HI-ChABC), and ChABC-injected mouse brains.

(C) Bar graphs showing CS stub 2B6 fluorescence intensity in ChABC-injected mice brains in the targeted CA2 area, proximal, and distal to the injection sites (rectangles in A).

(D-E) Bar graphs showing WFA fluorescence intensity in brain regions proximal (D) and distal (E) to the PNase, HI-ChABC, and ChABC injected sites (rectangles in A).

Bar data in B-D indicates Mean ± SEM, and dots represent data points. n = 4 slices from 4 mice in each group in B-D. One-way ANOVA, Tukey’s multiple comparisons test; *p < 0.05, **p < 0.01, ***p < 0.001,****p < 0.0001, and ns p>0.05.


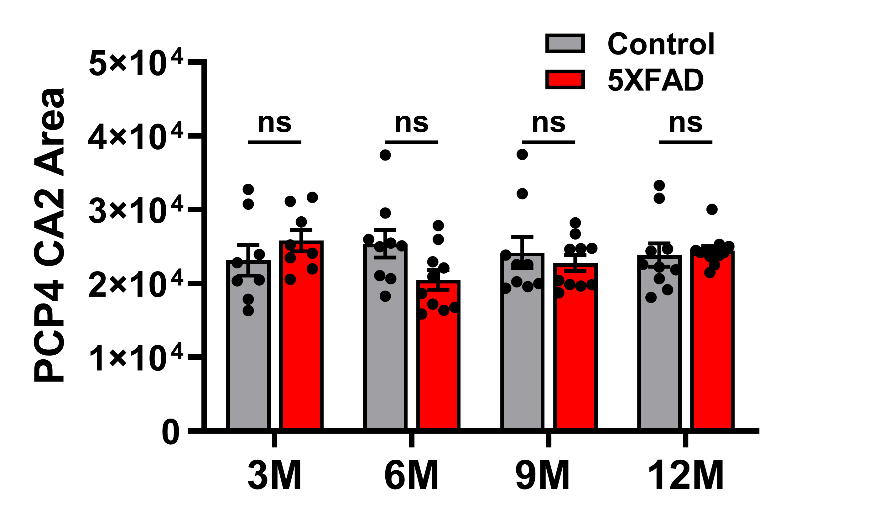
**Figure S2. Unaltered area of CA2 pyramidal neuron in control and 5XFAD mice in different age groups**

(A) Bar diagram showing the binary area occupied by the CA2 neuron marker PCP4 in different age groups in control and 5XFAD mice.

Bar data indicates Mean ± SEM, and dots represent data points. n = 8, 9, 9, 10 slices in 3, 6, 9, and 12M controls from 5 mice; n = 8, 10, 10, 12 slices in 3, 6, 9, and 12M 5XFAD from 5 mice. Two-way ANOVA, Šídák's multiple comparisons test; ns p>0.05.


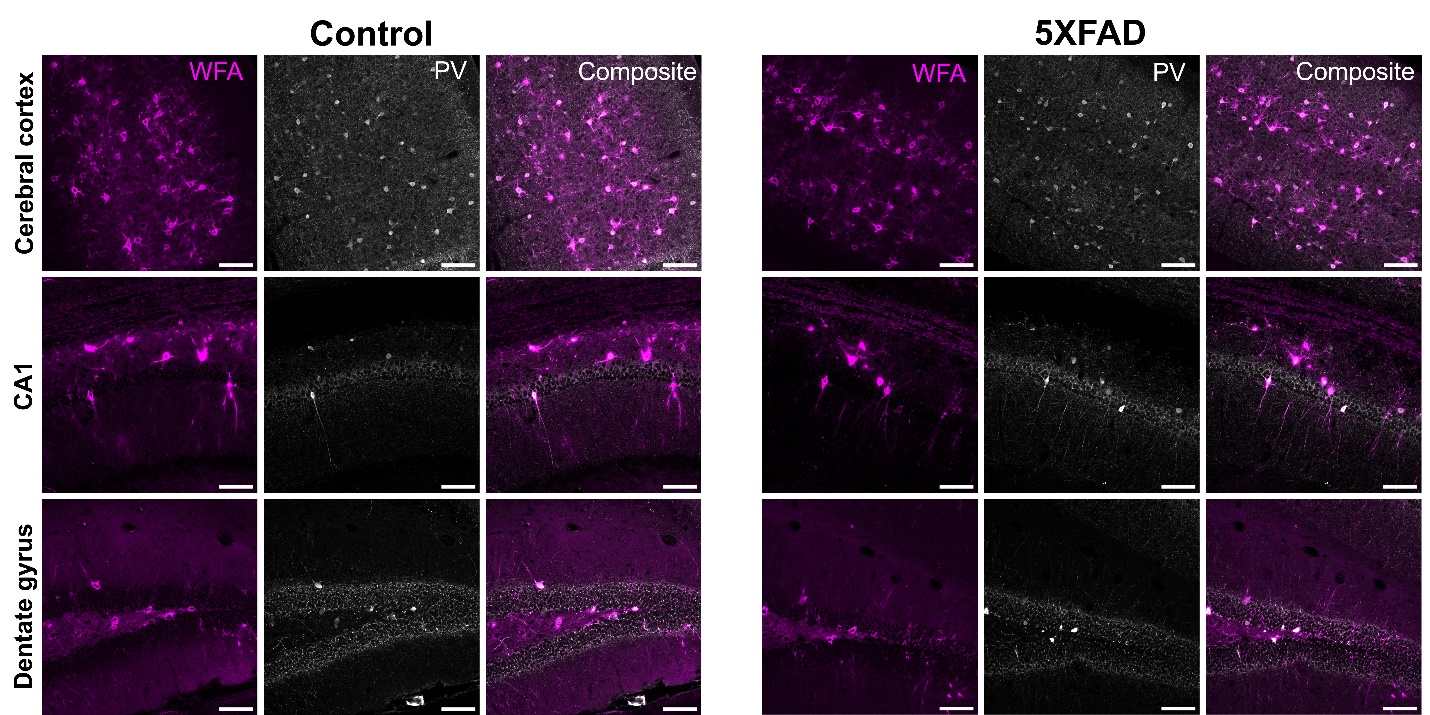


**Figure S3. Expression of PNNs on PV neurons in cortex and hippocampus in 5XFAD mice at 6 months of age**

Representative confocal micrographs showing immunohistochemical expression of WFA (magenta)-labeled PNNs around PV cells (gray) in the cerebral cortex, and CA1 and dentate gyrus areas of hippocampus from control and 5XFAD mice. Scale = 100µm.


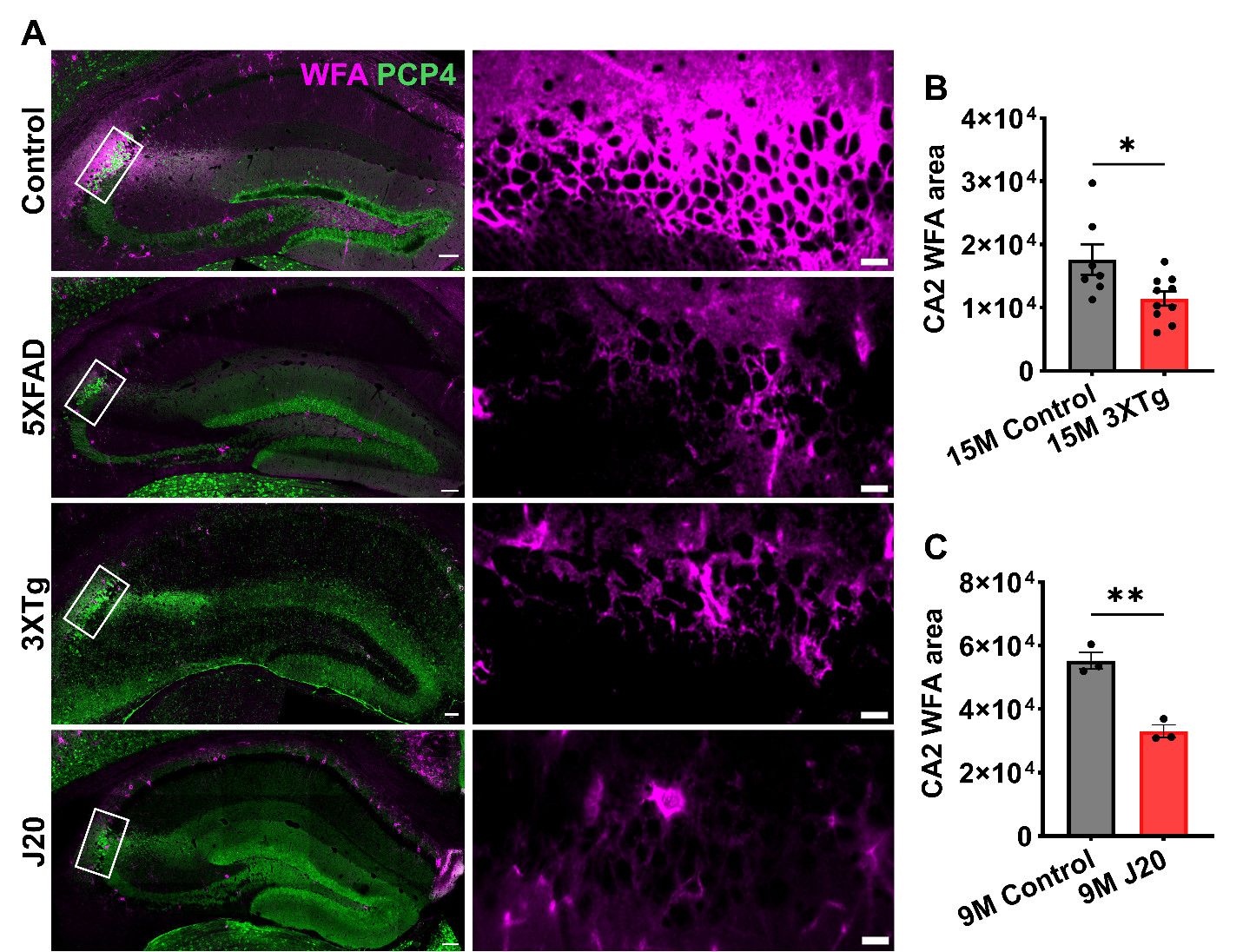


**Figure S4. PNN degradation occurs in multiple mouse models of AD**

Representative confocal micrographs showing WFA-labeled PNNs in entire hippocampi (left panel) and around CA2 pyramidal neurons (PCP4) in Control, 5XFAD, 3XTG, and J20 mice models of AD. White rectangular areas on CA2 are magnified in the right panel showing explicit disruption of CA2 PNNs in different models. Scale = 100µm left panel; 20µm right panel.

(B-C) Bar diagrams showing WFA area in CA2 in 3XTg (B) and J20 (C) models of AD.

Bar data indicates Mean ± SEM, and dots represent data points. n = 7 (Control) and 10 (3XTg) slices from 5 mice in both groups in B, n = 3 slices from 3 mice in both groups in C. unpaired two-tailed *t*-test; *p < 0.05, **p < 0.01.


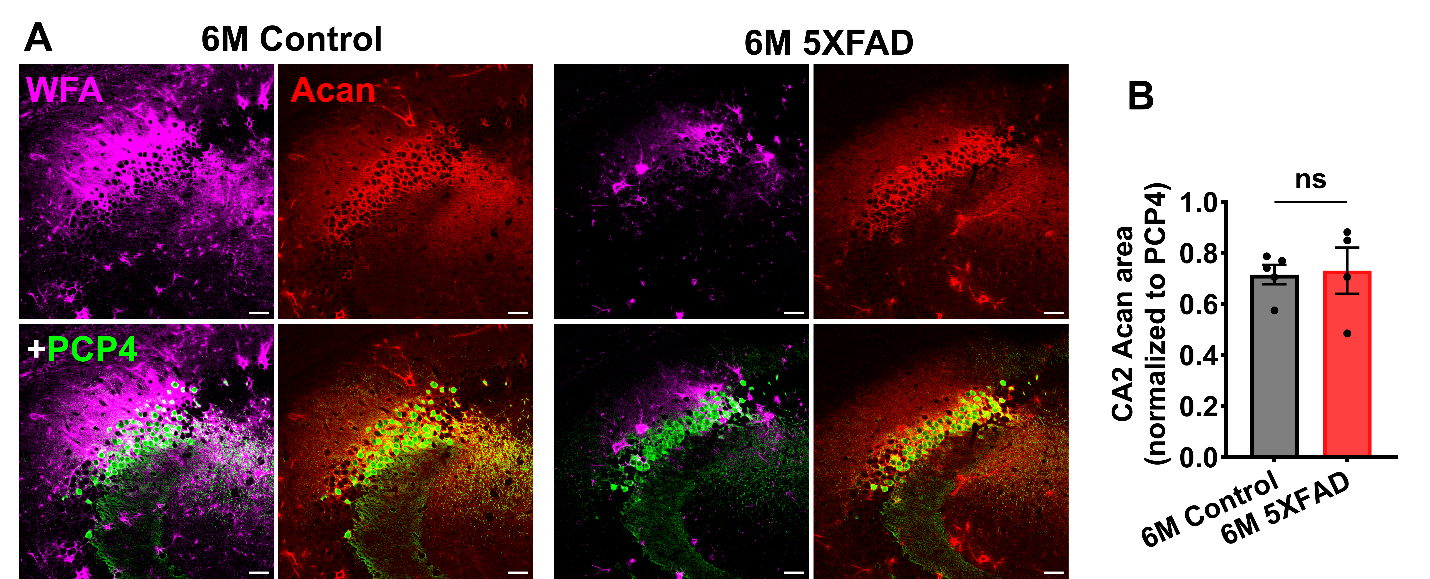


**Figure S5. Unaltered expression of aggrecan in CA2 area of 6M old 5XFAD mice**

(A) Representative confocal micrographs showing immunohistochemical labelling of WFA and Aggrecan-expressing PNNs in PCP4-labelled CA2 neurons in 6M old Control and 5XFAD mice. Scale 50µm.

(B) Bar diagrams showing Aggrecan occupied area in CA2 in 6M 5XFAD mice.

Bar data indicates Mean ± SEM, and dots represent data points. n = 5 slices from 5 mice (Control) and 4 slices from 4 mice (5XFAD). unpaired two-tailed *t*-test; ns p>0.05.


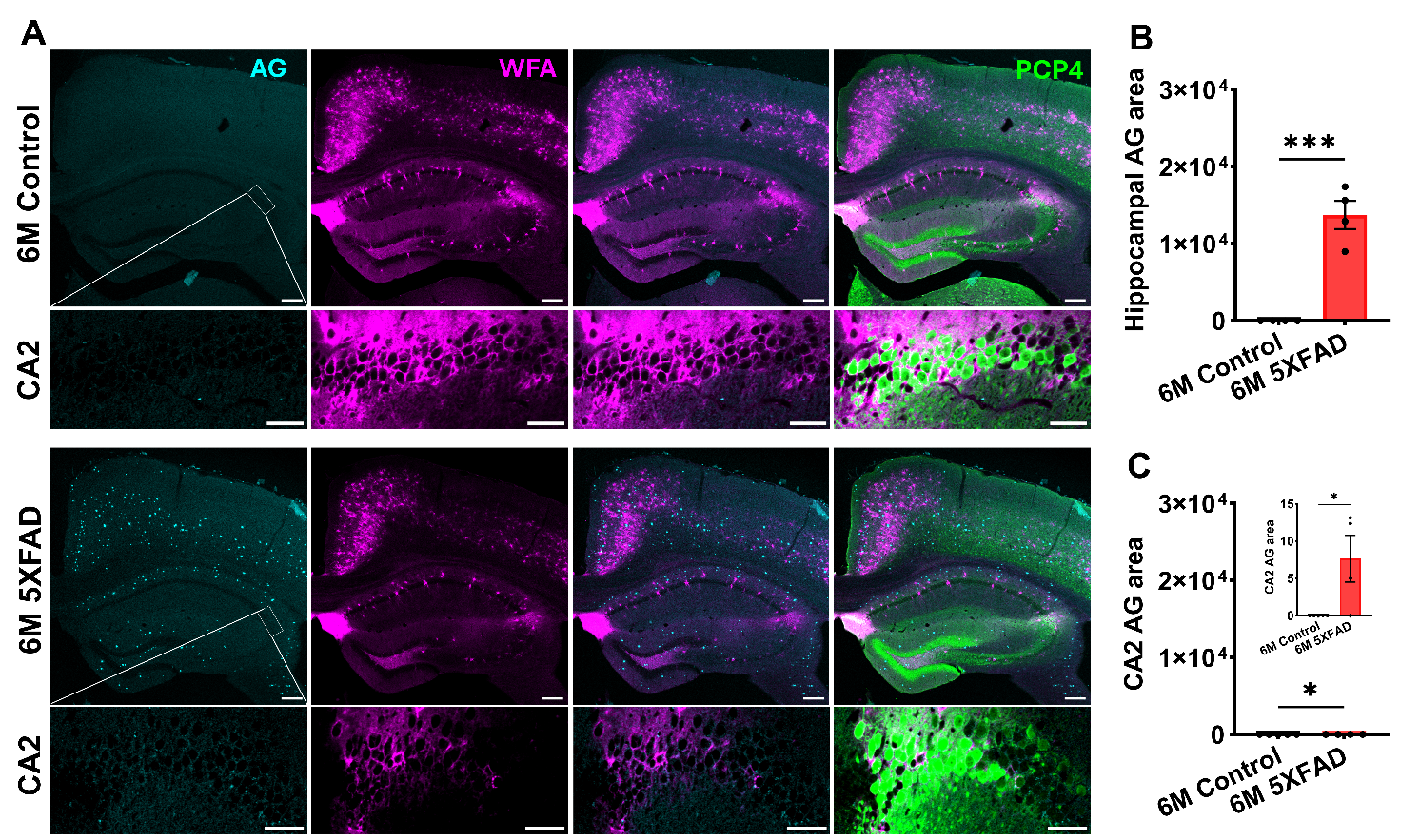


**Figure S6. Amyloid plaque in hippocampus of 6M old 5XFAD mice**

(A) Representative confocal micrographs showing immunohistochemical expression of markers of amyloid plaque AmyoGlo (AG), PNN (WFA), and CA2 neuron (PCP4) in 6M old 5XFAD mice. White rectangular areas on CA2 in the top panels are magnified in the bottom panels. Scale 200µm (main) and 50µm (magnified).

(B-C) Bar diagrams showing AG occupied area in the entire hippocampus (B) and in the CA2 area only (C). Graph C is magnified in the inset. Although statistically significant (inset graph), the AG area is minuscule in CA2 compared to the hippocampus (compare B and C with the same Y scale).

Bar data indicates Mean ± SEM, and dots represent data points. n = 5 slices from 3 mice (Control) and 4 slices from 5 mice (5XFAD) in B; n = 4 slices from 3 mice (Control) and 4 slices from 3 mice (5XFAD) in C. unpaired two-tailed *t*-test; *p < 0.05, ***p < 0.001.

**Figure S7. Training phase of novel recognition task showing preference for right or left objects by control and 5XFAD mice**

Bar diagram showing % Time spent with the left and right objects by control and 5XFAD mice. Bar data indicates Mean ± SEM, and dots represent data points. n = 8 (Control) and 14 (5XFAD); two-way ANOVA mixed- effects Šídák's; *p < 0.05, ***p < 0.001.


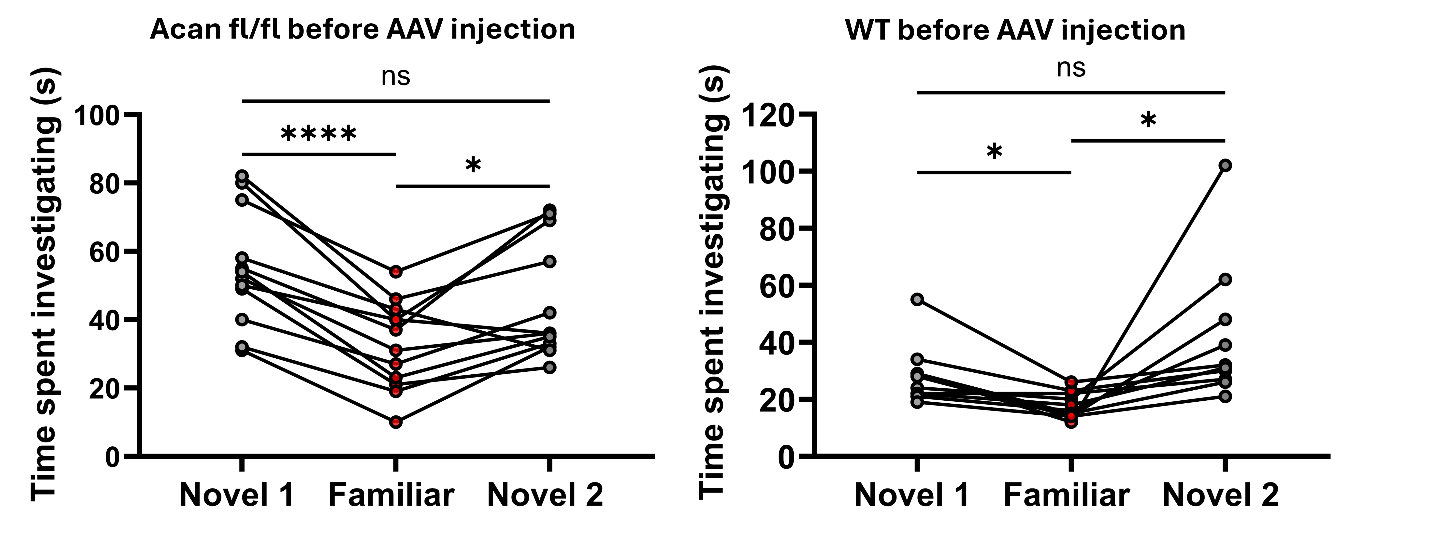


**Figure S8. Normal social memory in Acan fl/fl and WT mice before AAVCre injection**

Graphs showing normal social memory in Acan fl/fl (left) and WT (right) mice before AAV injecting AAV SynCre. n = 12 in Acan, 10 in WT group, one-way ANOVA, Tukey’s multiple comparisons test; *P < 0.05, **P < 0.01, ****P < 0.0001.


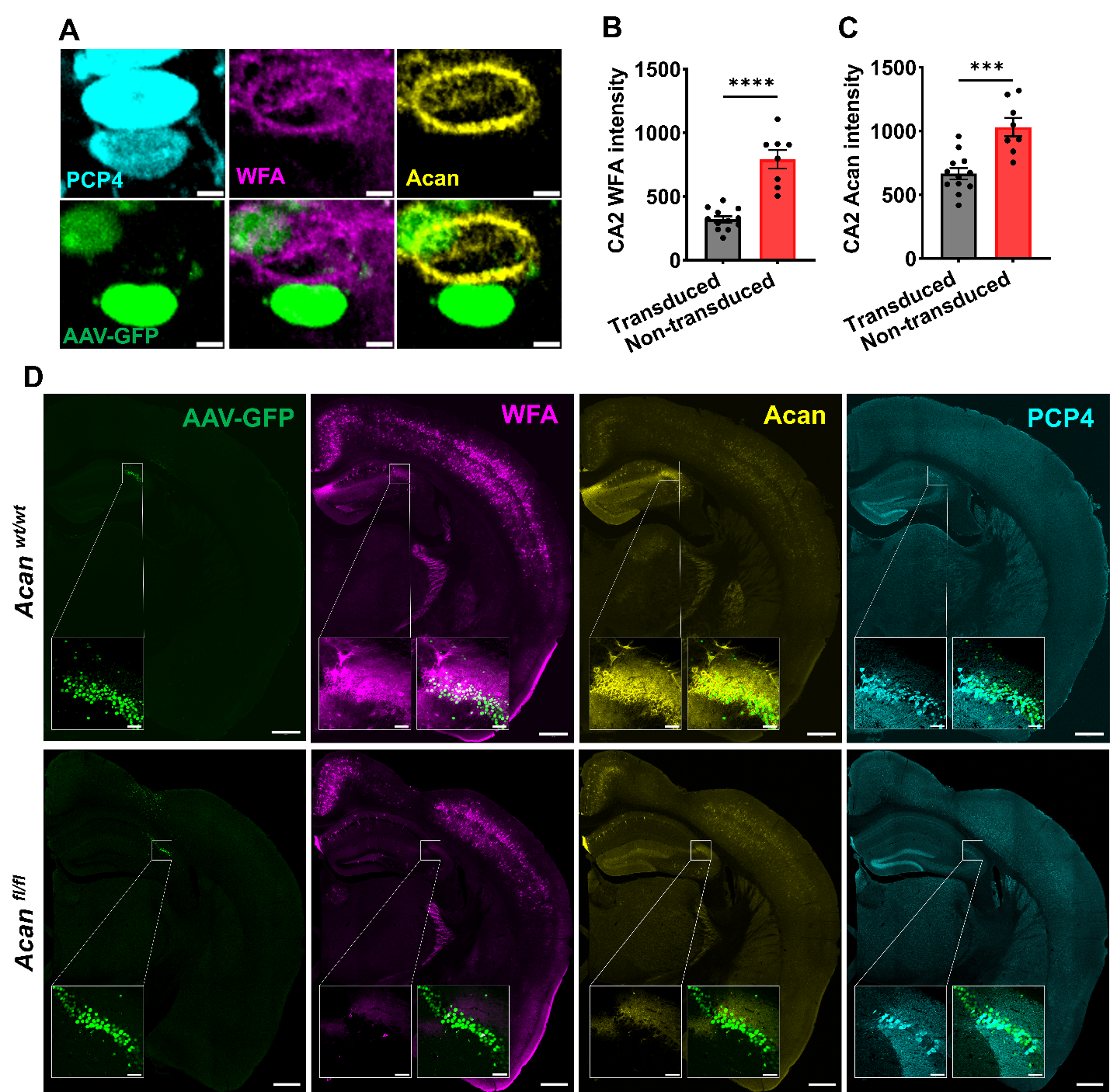


**Figure S9. AAVCre-mediated Aggrecan knockout eliminates PNNs in CA2 and surrounding brain areas**

(A) Confocal micrograph showing immunohistochemical expression of PNN markers WFA and Aggrecan (Acan) in AAVCre-transduced and non-transduced CA2 neurons (PCP4). No labelling of WFA and Acan in the CA2 neurons expressing GFP reporter (AAVGFP) confirms the knockout of PNNs. Scale 5µm.

(B-C) Bar diagrams showing fluorescence intensity of WFA (B) and Aggrecan (C) in AAV-transduced and non-transduced CA2 neurons. Bar data indicates Mean ± SEM, and dots represent data points. n = 12 (transduced) and 8 (non-transduced) neurons from 3 mice in both B and C. unpaired two-tailed *t*-test; ***p < 0.001, ****p < 0.0001.

(D) Confocal micrograph showing immunohistochemical expression of WFA and Acan-labelled PNNs and AAV reporter GFP in control (*Acan^wt/wt^*) and Aggreca KO (*Acan^fl/fl^*). The targeted CA2 area in the rectangle is magnified in the inset. The precision of AAV injection is reflected by major expression of GFP reporter in CA2 and a minimal spread in the cortex and hippocampal areas flanking CA2. Scale 500µm (main) and 50µm (magnified).
